# Plasma receptor interacting protein kinase-3 levels are associated with acute respiratory distress syndrome in sepsis and trauma: a cohort study

**DOI:** 10.1186/s13054-019-2482-x

**Published:** 2019-06-28

**Authors:** Michael G. S. Shashaty, John P. Reilly, Hilary E. Faust, Caitlin M. Forker, Caroline A. G. Ittner, Peggy X. Zhang, Meghan J. Hotz, David Fitzgerald, Wei Yang, Brian J. Anderson, Daniel N. Holena, Paul N. Lanken, Jason D. Christie, Nuala J. Meyer, Nilam S. Mangalmurti

**Affiliations:** 1 Pulmonary, Allergy, and Critical Care Division, Perelman School of Medicine, University of Pennsylvania, 5039 W Gates Building, 3600 Spruce Street, Philadelphia, PA 19104 USA; 2 Center for Clinical Epidemiology and Biostatistics, Perelman School of Medicine, University of Pennsylvania, Pennsylvania, USA; 3 Center for Translational Lung Biology, Perelman School of Medicine, University of Pennsylvania, Pennsylvania, USA; 4 Division of Traumatology, Surgical Critical Care, and Emergency Surgery, Perelman School of Medicine, University of Pennsylvania, Pennsylvania, USA

**Keywords:** Necroptosis, Sepsis, Trauma, Acute respiratory distress syndrome, Acute kidney injury

## Abstract

**Background:**

Necroptosis, a form of programmed cell death mediated by receptor interacting serine/threonine-protein kinase-3 (RIPK3), is implicated in murine models of acute respiratory distress syndrome (ARDS). We hypothesized that plasma RIPK3 concentrations in sepsis and trauma would be associated with ARDS development and that plasma RIPK3 would reflect changes in lung tissue RIPK3 in a murine model of systemic inflammation.

**Methods:**

We utilized prospective cohort studies of critically ill sepsis (*n* = 120) and trauma (*n* = 180) patients and measured plasma RIPK3 at presentation and 48 h. Patients were followed for 6 days for ARDS by the Berlin definition. We used multivariable logistic regression to determine the association of plasma RIPK3 with ARDS in each cohort, adjusting for confounders. In mice, we determined whether plasma and lung tissue RIPK3 levels rise concomitantly 4 h after injection with lipopolysaccharide and ZVAD-FMK, an apoptosis inhibitor.

**Results:**

The change in plasma RIPK3 from presentation to 48 h (ΔRIPK3) was associated with ARDS in sepsis (OR 1.30, 95% CI 1.03–1.63, per ½ standard deviation) and trauma (OR 1.79, 95% CI 1.33–2.40). This association was not evident for presentation RIPK3 levels. Secondary analyses showed similar findings for the association of ΔRIPK3 with acute kidney injury and 30-day mortality. Mice injected with lipopolysaccharide and ZVAD-FMK had significantly higher plasma (*p* < 0.001) and lung (*p* = 0.005) RIPK3 than control mice.

**Conclusions:**

The change in plasma RIPK3 from presentation to 48 h in both sepsis and trauma patients is independently associated with ARDS, and plasma RIPK3 may reflect RIPK3 activity in lung tissue.

**Electronic supplementary material:**

The online version of this article (10.1186/s13054-019-2482-x) contains supplementary material, which is available to authorized users.

## Background

Critical illness precipitated by sepsis or trauma is characterized by a dysregulated immune response that may result in organ dysfunction and consequent death or long-term morbidity [1–4]. Despite strides in early management strategies [5, 6], organ dysfunction syndromes such as the acute respiratory distress syndrome (ARDS) often complicate the early clinical trajectory of these patients and pose a significant barrier to improving outcomes. Disappointing results of trials for immune-targeted and other pharmacologic therapies have prompted interest in better understanding clinically relevant molecular pathways in ARDS through human translational study [7].

Necroptosis, a caspase-independent form of regulated cellular necrosis mediated by receptor interacting serine/threonine-protein kinase-3 (RIPK3) and mixed lineage kinase domain-like protein (MLKL) [8, 9], has recently been implicated as a key cell death modality in tissue and animal models of organ injury [10–13]. Studies by our group and others have shown that lipopolysaccharide- and red blood cell-induced lung injury is attenuated by inhibition of necroptosis [10, 13]. This form of cell death could have particular relevance to ARDS. Unlike apoptosis, necroptosis results in plasma membrane rupture and release of damage-associated molecular patterns (DAMPs) that drive tissue injury; distinct from conventional descriptions of necrosis, necroptosis induction and execution is highly regulated [9, 14–18]. Therefore, necroptosis may represent a novel potential therapeutic target for prevention or treatment of acute organ dysfunction.

Few studies to date have investigated necroptosis activation in critically ill patients. After finding that RIPK3 was released by cultured lung endothelial cells undergoing red blood cell-induced necroptosis, we reported an association of plasma RIPK3 with RBC transfusions and mortality in 37 sepsis patients [13]. We subsequently showed that plasma RIPK3 measured 48 h after presentation was associated with RBC transfusions and acute kidney injury (AKI) in 80 trauma patients [19]. Recent reports in medical ICU populations described higher plasma RIPK3 levels in mechanically ventilated and non-surviving patients [20, 21]. These studies provided limited information on the relationship of plasma RIPK3 with ARDS. Further, it remains unclear whether plasma RIPK3 is an accurate marker of expression of RIPK3 or execution of necroptosis in injured organs.

We sought to address these knowledge gaps using cohorts of critically ill sepsis and trauma patients as well as in vivo animal experiments. We hypothesized that plasma RIPK3 levels would be associated with ARDS in both sepsis and trauma populations, independent of patient-level characteristics. We also hypothesized that in a mouse model of systemic inflammation, plasma RIPK3 levels would correlate with lung tissue expression of the necroptosis mediators RIPK3 and MLKL. Secondarily, we sought to build on our prior reports [13, 19] by determining the association of plasma RIPK3 levels with AKI and mortality in larger cohorts of sepsis and trauma patients and by determining patient characteristics associated with plasma RIPK3. We studied these populations based on two considerations: first, sepsis and trauma are common critical illness syndromes with high rates of organ dysfunction; and second, sepsis and trauma are both characterized by a dysregulated immune response, ischemia-reperfusion injury, and treatment with blood product transfusions, all relevant for translation of existing pre-clinical studies of necroptosis [12, 13, 16, 18].

## Methods

Detailed descriptions of human cohort and experimental animal studies, including the STROBE checklist, are in Additional file 1. Key methods are summarized here.

### Sepsis and trauma cohorts

The Molecular Epidemiology of SepsiS in the Intensive care unit (MESSI) cohort and the PEnn TRauma Organ dysfunction Study (PETROS) are prospective cohort studies of critically ill sepsis and trauma patients, respectively, at the University of Pennsylvania [3, 22–24]. We included patients presenting to the emergency department and admitted to the medical ICU (MESSI, 2012–2014) or Penn Level I Trauma Center ICU (PETROS, 2012–2015) with plasma samples available at presentation and approximately 48 h later (*Plasma collection*, below). MESSI patients met American College of Chest Physicians/Society of Critical Care Medicine consensus criteria for severe sepsis or septic shock [25]. In PETROS, key exclusions were injury severity score (ISS) < 16 or death within 24 h of admission. Both studies were approved by the University of Pennsylvania Institutional Review Board.

### Data collection and outcomes

Clinical data were collected by medical record review. After prospective enrollment of patients, trained research personnel used REDCap-based electronic case report forms [26] to collect detailed medical history, physiologic variables, lab results, and treatment variables. We used the Berlin definition, with direct radiograph review by investigators, to identify incident ARDS and classify it as mild, moderate, or severe over the 6 days following presentation [27]. Patients were only considered to have ARDS if they were invasively mechanically ventilated at the time that they met ARDS criteria. We defined AKI by Acute Kidney Injury Network (AKIN) creatinine and renal replacement therapy (RRT) consensus criteria over the same time period [3, 28]. Mortality was determined at 30 days after admission.

### Plasma collection and RIPK3 measurement

We utilized blood samples drawn for clinical purposes at presentation to the emergency department and approximately 48 h after presentation. Samples were centrifuged within 30 min and refrigerated at 4 °C. Plasma aliquots were frozen within 12–48 h. We used enzyme-linked immunosorbent assay (Cusabio) to measure plasma RIPK3 concentrations [13, 19]. Plasma concentrations below the limit of detection (15.6 pg/ml) were set to 15.6 pg/ml for statistical analysis. Because our prior studies showed low RIPK3 levels at presentation in most trauma patients [19], and in order to account for RIPK3 release in the early stages of critical illness, we used the change in plasma RIPK3 from presentation to 48 h (ΔRIPK3) for primary analyses.

### Statistical analysis

In primary analyses, by cohort (MESSI, PETROS), we tested unadjusted associations of patient characteristics with ARDS using Student’s *t*, Wilcoxon rank-sum, *χ*^2^, or Fisher’s exact tests. We used multivariable logistic regression models to test the associations of ΔRIPK3 with ARDS adjusted for confounders. To avoid overfitting, we limited explanatory variables to approximately one for every ten outcomes [29]. We pre-specified these variables by cohort (Additional file 1) based on previously described associations with ARDS or RIPK3 [13, 19, 30]. We used post-estimation marginal analysis to determine adjusted risk of ARDS across a range of plasma ΔRIPK3 levels [31].

We repeated the primary ΔRIPK3-ARDS analyses stratified by several pre-specified patient characteristics (Additional file 1) and tested for interaction using likelihood ratio tests. In secondary analyses, we tested associations of ΔRIPK3 with AKI and mortality, associations of baseline characteristics with ΔRIPK3, and differences in ΔRIPK3 by organ dysfunction categories: ARDS alone, AKI alone, or both. Further secondary analysis details and sample size estimations are in Additional file 1. We used Stata/IC 13.1 (StataCorp, College Station, TX) and considered a two-tailed *p* < 0.05 significant for all analyses.

### Experimental animal studies

All experimental procedures were performed on 8–12-week-old female mice and conducted in accordance with the Institutional Animal Care and Use Committee at the University of Pennsylvania. Full details are in Additional file 1.

Mice were injected via tail vein with 10 mg/kg LPS (List Labs) as well as 10 mg/kg of the pan-caspase inhibitor ZVAD-FMK (BD Biosciences) in order to inhibit apoptosis and sensitize cells to necroptosis as previously described [16, 32–35]. Four hours following LPS-ZVAD administration, mice were sacrificed and both plasma and whole lungs were obtained for analysis. Plasma RIPK3 was measured using a murine ELISA kit (Cusabio). Lungs were homogenized and proteins were resolved by SDS-PAGE under reducing conditions. Immunoblotting was performed for RIPK3, MLKL, and phosphorylated MLKL normalized to β-actin. We determined differences in plasma and tissue RIPK3 between control and LPS groups using the Wilcoxon rank-sum test and tested the Spearman rank correlation between plasma and tissue RIPK3 (Stata/IC 13.1).

## Results

### MESSI cohort

From May 2012 to October 2014, 120 patients admitted from the emergency department were enrolled in the MESSI cohort and had plasma available at presentation and 48 h. Cohort characteristics are shown in Table 1. Septic shock was present before ICU admission in 78%, and the most common source of sepsis was pulmonary. ARDS developed in 44 (37%) patients and AKI in 41 (34%). Death at 30 days was more common in those with ARDS (55.6% vs. 33.3% for no ARDS, *p* = 0.017) or AKI (53.7% vs. 37.1% for no AKI, *p* = 0.090). ARDS had a maximum severity of mild in 6 (14%), moderate in 22 (50%), and severe in 16 (36%) cases.

**Table 1:** Baseline patient characteristics of patients in MESSI and PETROS cohorts

	MESSI cohort (*n* = 120)	PETROS cohort (*n* = 180)
Demographics		
Age, years	61 (50–68)	41 (25–62)
Male sex	71 (59)	140 (78)
Race^*a*^		
White	63 (53)	76 (42)
Black	51 (43)	89 (49)
Other^*b*^	6 (4)	15 (8)
Body mass index (kg/m^2^)	26.5 (22.1–32.7)	25.3 (22.7–28.5)
Medical history		
Hypertension^*a*^	70 (58)	50 (28)
Diabetes mellitus^*a*^	45 (38)	13 (7)
Congestive heart failure^*a*^	21 (18)	7 (4)
Chronic kidney disease^*ac*^	20 (17)	5 (3)
Chronic lung disease^*a*^	15 (13)	0 (0)
Chronic alcohol abuse^*ad*^	15 (13)	10 (6)
Smoking history^*ad*^		
Never	56 (47)	66 (40)
Former	27 (23)	25 (15)
Current	19 (16)	72 (44)
Acute injury and illness		
Blunt trauma mechanism	N/A	137 (76)
Injury Severity Score	N/A	25 (19–30)
Operation prior to ICU admission	N/A	74 (41)
APACHE II	29 (21–37)	17 (12–24)
Shock prior to ICU admission^*ae*^	93 (78)	89 (50)
Crystalloid, liters^*adf*^	3.5 (2.0–6.9)	2.5 (1.2–4.0)
Pulmonary source of sepsis	51 (43)	N/A
Transfusions (days 0 + 1)^*g*^		
Received RBC transfusion	31 (26)	108 (60)
Number of units	2 (1–2)	5 (3–9)
Received FFP transfusion	16 (13)	67 (37)
Number of units	3 (2–4)	4 (2–6)
Received platelet transfusion	18 (15)	62 (34)
Number of doses^*h*^	2 (1–3)	2 (1–2)
Outcomes		
ARDS	44 (37)	37 (21)
AKI^*i*^	41 (37)	53 (30)
30-day mortality	50 (42)	17 (9)

Data are shown as *n* (%) for categorical variables and median (interquartile range) for continuous variables. Definition of abbreviations: *ARDS* acute respiratory distress syndrome, *SBP* systolic blood pressure, *ED* emergency department, *ICU* intensive care unit, *RBC* red blood cell, *FFP* fresh frozen plasma. ^*a*^Missing data for PETROS cohort: race (*n* = 4); hypertension [4]; diabetes [3]; coronary heart disease [3]; congestive heart failure [2]; chronic kidney disease [1]; chronic lung disease [1]; chronic alcohol abuse [5]; smoking history [17]; shock prior to ICU admission [1]; crystalloid [4]. ^*b*^In PETROS cohort, Asian (*n* = 9), North American Indian/Alaskan Native (n = 2), and Unknown (n = 2); in MESSI cohort, Asian (n = 1) and unknown (*n* = 5). ^*c*^Includes patients with end-stage renal disease (MESSI *n* = 9, PETROS n = 1). ^*d*^Missing data for MESSI cohort: chronic alcohol abuse (*n* = 19); smoking history [18]; crystalloid [2]. ^*e*^Shock defined as need for vasopressors or mean arterial pressure < 65 mmHg (MESSI) or systolic arterial pressure < 90 mmHg (PETROS). ^*f*^Administered during the first 24 h after ED presentation (MESSI) or prior to ICU arrival (PETROS). ^*g*^The calendar day of and the day after presentation. ^*h*^Each platelet dose at our institution is roughly equivalent to 4 single-donor platelet units or 6 pooled platelet units. ^*i*^AKI numbers exclude patients with end-stage renal disease

### PETROS cohort

From April 2012 to January 2015, 180 patients were enrolled in the PETROS cohort and had plasma available at presentation and 48 h (Table 1). The median ISS was 25 (19–29.5) and 137 (76.1%) had blunt trauma mechanism. The median age was 20 years younger than in MESSI, 140 (79%) were male, and blood product transfusions were far more common than in MESSI. ARDS occurred in 37 (20.6%) patients and AKI in 53 (29.6%). Mortality was much higher in patients who developed ARDS (29.7% vs. 4.2% for no ARDS, *p* < 0.001) or AKI (17.0% vs. 5.6% for no AKI, *p* = 0.014). ARDS had a maximum severity of mild in 7 (19%), moderate in 21 (57%), and severe in 9 (24%) cases.

### Plasma RIPK3 and ARDS in human cohorts

In both cohorts, patients who developed ARDS during the first 6 days had a significantly greater increase in plasma RIPK3 concentration from presentation to 48 h (ΔRIPK3) than those who did not develop ARDS (Table 2). Findings were similar for plasma RIPK3 at 48 h. There was no association, however, between day 0 plasma RIPK3 levels and ARDS. Of note, 103 (57.2%) trauma patients had day 0 levels below the limit of detection.

**Table 2:**
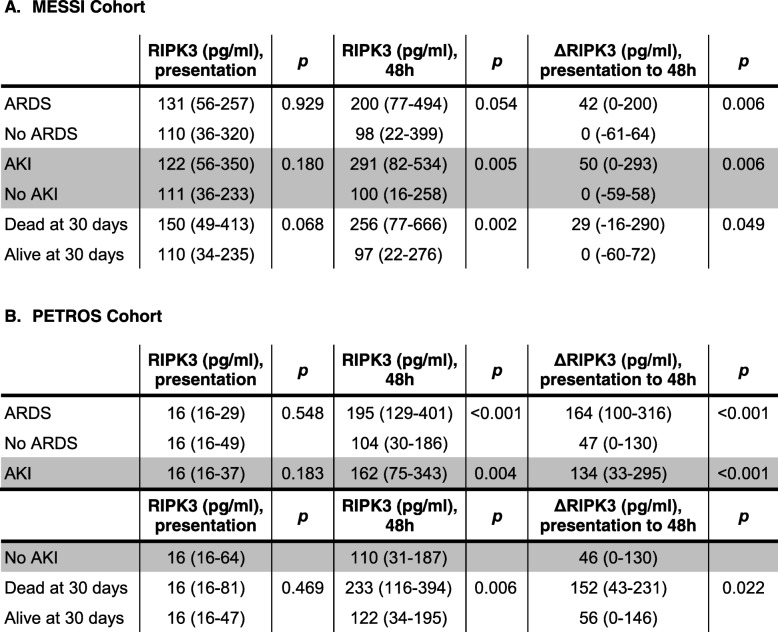
Unadjusted associations of plasma RIPK3 concentrations with organ dysfunction and death

All *p* values are from comparisons using the Wilcoxon rank-sum test. Analyses involving AKI exclude patients with end-stage renal disease (MESSI cohort *n* = 9; PETROS cohort *n* = 1). Definition of abbreviations: *ARDS* acute respiratory distress syndrome, *AKI* acute kidney injury, *RIPK3* receptor interacting protein kinase-3

**Fig. 1:**
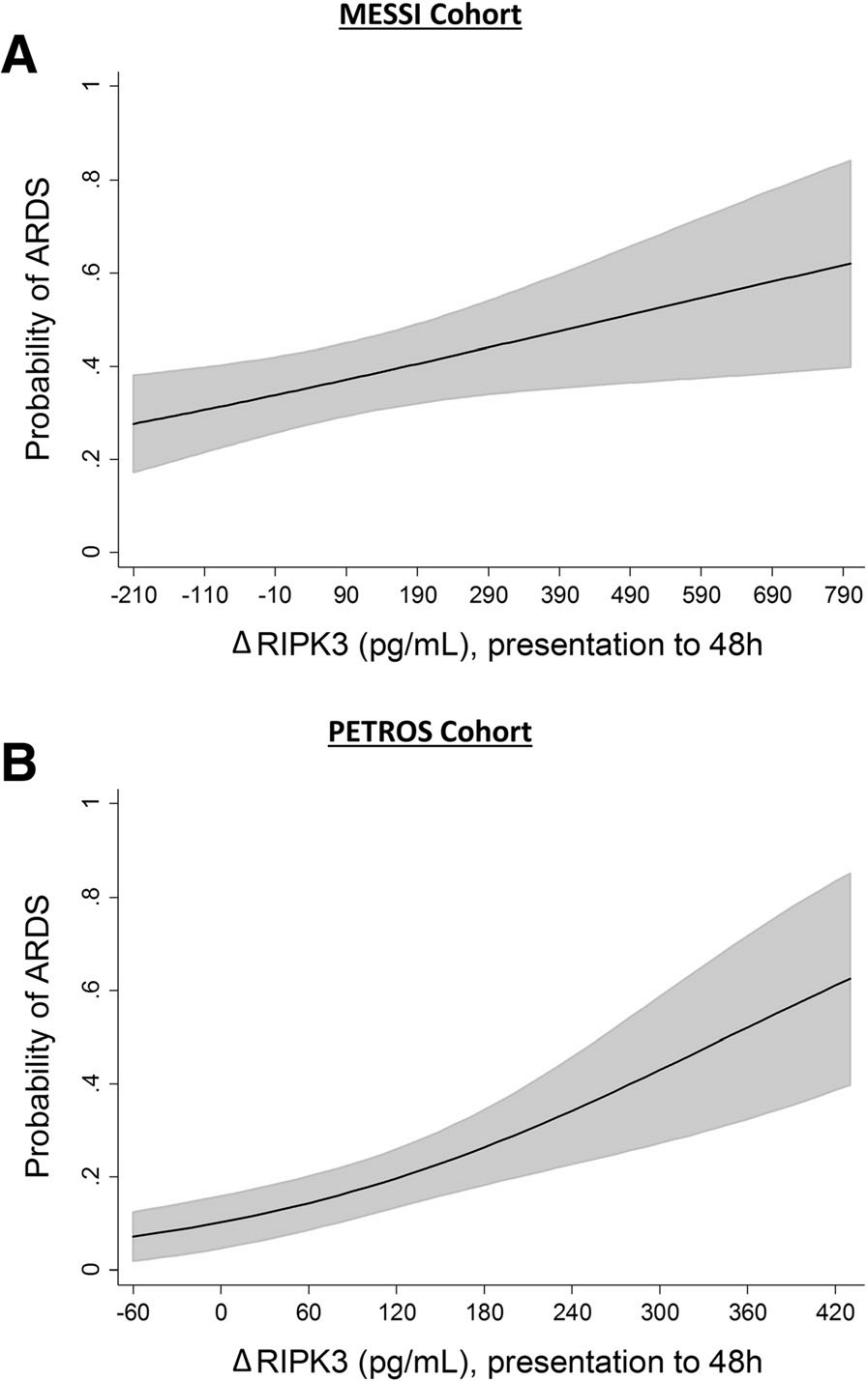
Adjusted probability of acute respiratory distress syndrome (ARDS) across the range of ΔRIPK3 (change from presentation to 48 h) in each cohort. Estimated probabilities (line) with 95% confidence intervals (gray shading) determined using post-estimation marginal analysis after multivariable logistic regression modeling. **a** MESSI cohort, probabilities adjusted for age, red blood cell transfusions on day of presentation, lung source of sepsis, and shock at presentation. **b** PETROS cohort, probabilities adjusted for red blood cell transfusions in the first 6 h, trauma mechanism, and injury severity score

The association of ΔRIPK3 with ARDS remained significant in both cohorts in multivariable regression models adjusting for potential confounders (Table 3). Based on these models, Fig. 1 shows that the adjusted risk of ARDS increased from 30 to > 60% across the range of ΔRIPK3 in MESSI (1a) and from < 10% to 60% across the range of ΔRIPK3 in PETROS (1b).

**Table 3:**
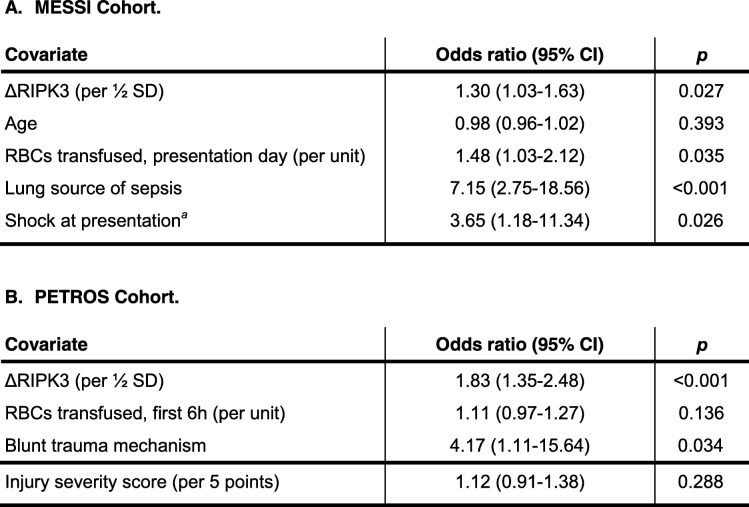
Multivariable logistic regression models of the ΔRIPK3-ARDS association adjusted for pre-specified confounders

For A. and B.: ΔRIPK3 remains significantly associated with ARDS after adjustment for pre-specified confounders. The odds ratio corresponds to the adjusted association of each covariate with ARDS. ^*a*^Shock defined as need for vasopressors or mean arterial pressure < 65 mmHg. Definition of abbreviations: *ARDS* acute respiratory distress syndrome, *RIPK3* receptor interacting protein kinase-3, *SD* standard deviation, *RBCs* red blood cells

In MESSI, the association of ΔRIPK3 with ARDS was similar when patients were stratified by infection source (adjusted OR 1.31 (95% CI 0.88–1.97) for pulmonary (*n* = 51); 1.30 (0.98–1.72) for non-pulmonary (*n* = 69); interaction *p* = 0.844) and did not differ significantly by shock (adjusted OR 1.23 (0.97–1.56) if present (*n* = 93); 2.79 (1.00–7.81) if absent (*n* = 27); interaction *p* = 0.287). In PETROS, the association of ΔRIPK3 with ARDS was similar when patients were stratified by trauma mechanism (adjusted OR 1.93 (1.39–2.69) for blunt trauma (*n* = 137); 1.96 (1.16–3.34) for penetrating trauma (*n* = 43); interaction *p* = 0.958).

We further analyzed whether ΔRIPK3 elevations might precede ARDS by comparing patients without ARDS to only those who developed ARDS after the first 48 h (the second time point of RIPK3 testing). In PETROS, 18/37 (48.6%) ARDS patients developed ARDS after 48 h. These patients had significantly higher ΔRIPK3 levels than those without ARDS (median 163.7 pg/ml vs. 48.9 pg/ml, respectively, *p* < 0.001, Additional file 2: Figure S1). In MESSI, there were too few patients who developed ARDS after 48 h (*n* = 5) to conduct a similar subgroup analysis.

Because phenytoin may inhibit necroptosis [36], we examined the impact of phenytoin use on ΔRIPK3 and its relationship with ARDS. In PETROS, the 27/180 (15%) patients who received phenytoin within the first 48 h had lower median ΔRIPK3 levels than those who did not (36.7 (IQR 0–104.8) pg/ml vs. 68.5 (0–178.5) pg/ml, respectively), though the difference was not statistically significant (*p* = 0.071). Adding phenytoin to the primary multivariable model, however, minimally changed the association of ΔRIPK3 and ARDS (OR 1.83 (95% CI 1.35–2.49), *p* < 0.001). Only two MESSI patients received phenytoin in the first 48 h, precluding similar analyses in this cohort.

### Plasma and lung RIPK3 in mouse model

**Fig. 2:**
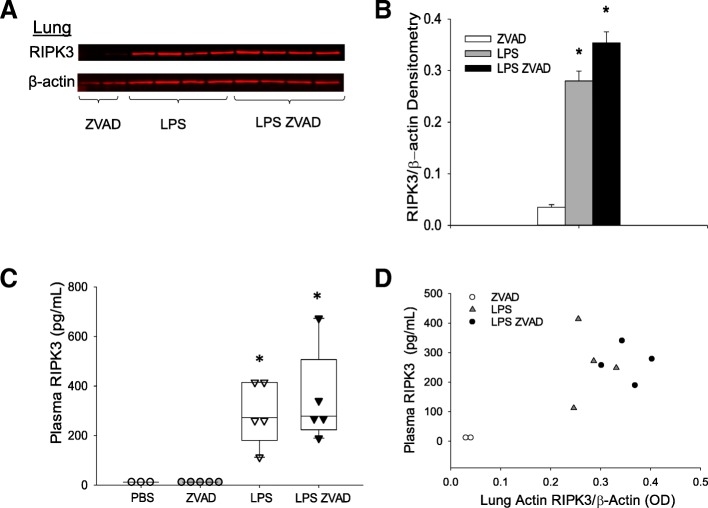
Lung and plasma RIPK3 are elevated following LPS and LPS-ZVAD treatment. **a** Whole lung homogenate of mice 4 h following LPS, LPS-ZVAD treatment; *n* = 2 studies (5–8 mice/group), immunoblot from one study is shown. **b** Densitometry of whole lung homogenate, *p = 0.016 LPS vs. ZVAD, *p* = 0.005 LPS-ZVAD vs. ZVAD. **c** Plasma RIPK3, *p = 0.002 LPS vs. PBS or ZVAD, *p* = 0.002 LPS-ZVAD vs. PBS or ZVAD. **d** Correlation of lung tissue and plasma RIPK3 (Spearman’s *ρ* = 0.55, *p* = 0.102)

It has previously been reported that lung RIPK3 and MLKL are elevated following intratracheal LPS administration [10]. We asked whether lung RIPK3 and MLKL expression are elevated following systemic LPS, which induces a systemic inflammatory state relevant to human sepsis and trauma, and whether plasma RIPK3 shows a concomitant rise. Both lung RIPK3 expression (Fig. 2a, b) and plasma RIPK3 concentration (Fig. 2c) were significantly elevated following LPS or LPS in the presence of the pan-caspase inhibitor ZVAD-FMK when compared with PBS or ZVAD-FMK alone. There was a positive correlation between lung and plasma RIPK3 (Fig. 2d), though this correlation did not reach statistical significance (Spearman’s *ρ* = 0.55, *p* = 0.102). We did not observe increased lung MLKL or phosphorylated MLKL expression following systemic LPS (Additional file 2: Figure S2).

### Secondary analyses

In MESSI, ΔRIPK3 and 48-h RIPK3 were significantly higher in patients who developed AKI than those who did not (Table 2). This association was also present in 180 PETROS patients, which included 80 patients in whom we previously reported a RIPK3-AKI association [19]. These associations were independent of pre-specified confounders (Additional file 1: Table S1 and Additional file 2: Figure S3). In both cohorts, those with both AKI and ARDS had the highest ΔRIPK3 levels, though patients with AKI or ARDS only still had significantly higher ΔRIPK3 than those with neither (Additional file 2: Figure S4). The associations of ΔRIPK3 with either ARDS or AKI largely remained significant when adjusted for AKI or ARDS, respectively (Additional file 1: Table S2).

ΔRIPK3 plasma concentration was also associated with mortality in both cohorts (Table 2). In MESSI, this association remained significant after adjustment for presence of shock at presentation and other confounders (adjusted OR 1.27, 95% CI 1.03–1.57, *p* = 0.028; Additional file 1: Table S3).

Additional file 1 Table S4 shows the association of patient characteristics with ΔRIPK3 in each cohort. APACHE II score was strongly associated with ΔRIPK3 in both sepsis and trauma patients. ΔRIPK3 also increased with greater numbers of blood products, though this association was most notable in PETROS, in which transfusions were far more common. In PETROS, younger age, non-Caucasian race, penetrating trauma, shock prior to ICU admission, need for emergent operation, and increased crystalloid resuscitation were also associated with higher ΔRIPK3. Underweight and obese MESSI patients had higher ΔRIPK3 than normal and overweight patients, though these findings were not statistically significant (*p* = 0.073). As noted, the majority of PETROS patients had presentation RIPK3 levels below the limit of detection; only congestive heart failure and blunt trauma mechanism were associated with higher RIPK3 levels at this time point (Additional file 1: Table S5). In MESSI, however, younger age, chronic alcohol abuse, higher APACHE II score, and higher volume of crystalloid resuscitation were associated with presentation RIPK3 concentrations. In addition, median presentation RIPK3 was higher in patients who died by day 30, though this difference was not statistically significant (*p* = 0.068).

## Discussion

In this study, we demonstrated that the change in plasma RIPK3 concentration from presentation to 48 h was independently associated with ARDS in two major at-risk populations, sepsis and trauma and that both lung RIPK3 expression and plasma RIPK3 concentrations rose significantly in mice treated with systemic LPS alone or when given with an inhibitor of apoptosis. Despite a wealth of recent animal and tissue studies of necroptosis showing its potential relevance to multiple human syndromes including ARDS [9, 10, 15, 16], data in human populations remain limited [13, 19–21, 37]. Our study provides the largest analysis to date of RIPK3 in ARDS, takes steps toward understanding the role of plasma RIPK3 as a marker of lung injury, and strengthens prior findings that plasma RIPK3 was associated with AKI and mortality. In the context of existing preclinical data [10, 13], these findings collectively suggest that necroptosis and other RIPK3-regulated pathways may be mechanistically important in ARDS and other acute organ dysfunction syndromes.

Since its description in 2009 [38], RIPK3-mediated necroptosis has emerged as a key mechanism in preclinical models of acute lung and renal injury [10, 13, 15]. The characteristic release of tissue-injurious DAMPs during necroptosis makes it of great interest as a driver, and therefore potential therapeutic target, of acute organ injury [13, 34, 39–41]. In sepsis patients, Davenport et al. identified increased gene expression of *RIPK3* and *MLKL* in circulating leukocytes as part of a molecular response subtype characterized by a two- to threefold mortality increase [42]. There are now reports of plasma RIPK3 associated with mortality [13, 19, 21], AKI [19, 37], and mechanical ventilation [20]. Studies on RIPK3 in ARDS, however, are limited, the largest being a subgroup analysis that included 24 patients with ARDS [20]. In cohorts with over three times that number of ARDS cases, we now show a convincing association of plasma RIPK3 with ARDS independent of relevant confounders. We also add novel findings about the time course of the RIPK3-ARDS association. While the rise in plasma RIPK3 over the first 48 h was clearly able to distinguish ARDS from non-ARDS cases, there was no signal that RIPK3 on presentation to the ED or trauma bay could predict ARDS, with similar findings for AKI and mortality. These results have potential implications for clinical utility: by 48 h, when ARDS is often already manifest, this biomarker may be most helpful to identify a subgroup with RIPK3 activation for possible targeted treatment. In fact, RIPK3 inhibitors have already shown protection against tissue injury in preclinical studies [35, 43], and efforts to translate these findings into effective therapies may be aided by understanding patient groups most likely to respond. For consideration of RIPK3 as an ARDS prediction or prevention tool, however, studies of serial early measurements would be needed to determine how rapidly after presentation the RIPK3-ARDS association becomes evident.

For any such pathway-targeted applications, though, it is important to know to what degree plasma RIPK3 reflects underlying lung injury. We previously demonstrated that human vascular endothelial cells undergoing necroptosis release RIPK3 [13], but it remained unclear if in vivo circulating RIPK3 levels reflected RIPK3 expression in lung tissue. Our current finding that murine lung and plasma RIPK3 rose substantially and concomitantly in response to systemic LPS and LPS-ZVAD suggests that the RIPK3-ARDS association in sepsis and trauma patients could reflect increased expression and release of RIPK3 from injured lung tissue, injury that is not explained by apoptosis. While necroptotic cell death is one explanation for these findings, other RIPK3-dependent pathways may be involved: Lawlor et al. have shown that RIPK3 can promote inflammasome activation independent of MLKL and necroptosis [44]. Notably, we found that pMLKL, an intracellular mediator of necroptosis downstream of RIPK3, did not increase after LPS or LPS-ZVAD. These findings are also consistent with those of Siempos et al. in which RIPK3-deficient mice were protected from ventilator-induced lung injury while MLKL-deficient mice were not [20]. Further studies, potentially including testing of plasma pMLKL and other key cell death pathway mediators that may be involved in the RIPK3-ARDS association, are important if therapies targeting programmed necrosis are to be considered for acute lung injury.

Our study expands on smaller reports that plasma RIPK3 is associated with AKI in sepsis and trauma patients [19, 37]. We now demonstrate a RIPK3-AKI association robust to adjustment for relevant confounders and independent of ARDS. This lends further clinical relevance to multiple preclinical studies showing the importance of RIPK3 and necroptosis in acute renal injury [8, 12, 15, 37, 45]. It is highly plausible that programmed necrosis in the kidneys, as well as the lungs and other organs, results in elevated circulating RIPK3 levels in sepsis and trauma patients. It is also possible that circulating RIPK3 is itself a causal factor in multiple organ dysfunction, similar to well-established DAMPs like cell-free DNA. In either case, RIPK3 may identify a process of necroinflammation in which the release of a diverse groups of DAMPs by necrotic cells serve to propagate and sustain the inflammatory response [46]. For example, we previously found that the DAMP high-mobility group box 1 protein (HMGB1) released following transfusion-induced necroptosis primes mice to subsequent lung injury [13], and others showed that cigarette smoke-induced necroptosis and DAMP release increase airway inflammation [47]. The complexity of how necroptosis and other regulated necrosis pathways result in and interact with release of myriad DAMPs in vivo to promote inflammation, tissue injury, and multiple organ dysfunction remains inadequately understood. Clinically relevant animal models of sepsis and trauma may be best suited to clarify these knowledge gaps.

There are limited existing data on patient characteristics associated with RIPK3 levels [19, 20]. We found that severity of illness measures tracked with plasma ΔRIPK3 in both sepsis and trauma. In MESSI but not PETROS, this was true for presentation RIPK3 as well, possibly reflecting a greater delay from initial insult to ED presentation in sepsis patients, allowing more time for circulating RIPK3 to rise. In PETROS, variables including race, penetrating trauma, shock, crystalloid volume, and blood product transfusions were all significantly associated with ΔRIPK3. We have previously shown that RBCs can induce RIPK3 release from lung endothelial cells [13]. Transfused patients are also at increased risk of ARDS [30]. If RIPK3 proves to be a causal link, targeting RIPK3 pathways could be considered to reduce ARDS rates among the substantial number of trauma patients requiring transfusions.

Our study has several limitations. First, we did not have plasma samples at time points between presentation and 48 h. The kinetics of plasma RIPK3 in the early hours of sepsis and trauma remain unclear, as does the ability of RIPK3 at such time points to predict subsequent ARDS. While we showed in trauma patients that ΔRIPK3 was associated with ARDS developing after 48 h, future studies with serial RIPK3 measurements may provide more granular detail of the time-varying relationship of RIPK3 and ARDS during early critical illness to allow for more robust causal inference. Second, while our study is the largest analysis to date of RIPK3 and ARDS, our cohort sizes did not allow adjustment for all possible confounders without potentially overfitting the multivariable models. Third, while the significant rise in murine plasma RIPK3 concentrations in response to LPS and LPS-ZVAD mirrored that seen in lung tissue, larger studies would be needed to firmly establish a tight correlation of plasma and lung RIPK3 concentrations. Studies testing RIPK3 expression in other organs may help to determine whether plasma RIPK3 also reflects extra-pulmonary tissue expression. Fourth, the specificity of RIPK3 as a marker of necroptosis in the lung or other organs remains unknown in human populations. Future studies could include using tissue or fluid obtained from affected organs, such as bronchoalveolar lavage fluid or urine, to validate plasma RIPK3 as a non-invasive marker of necroptosis or other RIPK3-related pathways. Finally, whether clinically available tests reflecting cell death such as lactate dehydrogenase strongly correlate with RIPK3, and therefore could be used as surrogates, remains unclear but could be tested in future studies.

## Conclusions

We demonstrated a significant association of plasma ΔRIPK3 with ARDS in critically ill sepsis and trauma patients and showed that both lung and plasma RIPK3 increased rapidly in mice injected with LPS-ZVAD. These findings extend prior preclinical studies and suggest that necroptosis and other RIPK3-dependent processes may be important mechanisms underlying ARDS in these two at-risk populations. Ongoing studies of this marker may prove useful in identifying novel molecular pathways to target for ARDS prevention and treatment.

## Additional files

Additional file 1: Methods, STROBE statement, and supplemental tables. (DOCX 127 kb)

Additional file 2: **Figure S1.** Change in plasma RIPK3 concentration from presentation to 48 h by Acute Respiratory Distress Syndrome (ARDS) status excluding patients who met ARDS criteria ≤ 48 h after presentation among patients in the PETROS (trauma) cohort (No ARDS *n* = 143, ARDS *n* = 18 meeting criteria 55–140 h after presentation). Gray boxes represent interquartile range, with median designated by central line. **Figure S2.** Levels of mixed lineage kinase domain-like protein (MLKL) and phosphorylated MLKL (pMLKL) in whole lung homogenate are not significantly different between mice treated with ZVAD, LPS, or LPS-ZVAD. **A.** Immunoblot of whole lung homogenate showing similar MLKL and pMLKL regardless of treatment. **B.** Densitometry of whole lung homogenate. All comparisons between treatment groups are non-significant. **Figure S3.** Adjusted probability of acute kidney injury (AKI) across range of Δ receptor interacting protein kinase-3 (RIPK3) levels (RIPK3 change from presentation to 48 h) in each cohort. Estimated probabilities (line) with 95% confidence intervals (gray shading) determined using post-estimation marginal analysis after multivariable logistic regression modeling. **A.** MESSI cohort. Probabilities adjusted for age, red blood cell transfusions on the day of presentation, chronic kidney disease, diabetes mellitus, and shock at presentation. **B.** PETROS cohort. Probabilities adjusted for red blood cell transfusions in the first 6 h after presentation, trauma mechanism, abdominal injury severity, and shock prior to ICU admission. **Figure S4.** Plasma levels of Δ receptor interacting protein kinase-3 (ΔRIPK3, change from presentation to 48 h) by acute kidney injury (AKI) and acute respiratory distress syndrome (ARDS) category in human cohorts. Shaded portions of box plots show median concentrations (central line) and 25th and 75th percentiles (bottom and top lines of box). Brackets above the *p* values denote the two organ dysfunction categories being compared (Wilcoxon rank-sum test). **A.** MESSI cohort. Patients with AKI only, ARDS only, or AKI + ARDS had higher ΔRIPK3 than those with neither AKI nor ARDS. **B.** PETROS cohort. Patients with AKI + ARDS had higher ΔRIPK3 than those with AKI or ARDS only, groups which in turn had higher ΔRIPK3 than patients with neither. (ZIP 1021 kb)

## Data Availability

The authors will furnish deidentified copies of the analytic datasets used upon reasonable request, pending permission from the University of Pennsylvania Institutional Review Board.

## Abbreviations

AKI: Acute kidney injury
AKIN: Acute Kidney Injury Network
ARDS: Acute respiratory distress syndrome
DAMP: Damage-associated molecular pattern
ELISA: Enzyme-linked immunosorbent assay
HMGB1: High-mobility group box 1 protein
ISS: Injury Severity Score
LPS: Lipopolysaccharide
MESSI: Molecular Epidemiology of SepsiS in the ICU cohort
MLKL: Mixed lineage kinase domain-like protein
PETROS: PEnn TRauma acute Organ dysfunction Study
pMLKL: Phosphorylated MLKL
RBC: Red blood cell
RIPK3: Receptor interacting serine/threonine-protein kinase-3
RRT: Renal replacement therapy
SDS-PAGE: Sodium dodecyl sulfate polyacrylamide gel electrophoresis
ΔRIPK3: Change in RIPK3 concentration from presentation through approximately 48 h later
