# Supplementary material for: Plasma receptor interacting protein kinase-3 levels are associated with acute respiratory distress syndrome in sepsis and trauma: a cohort study
Source: Crit Care. 2019 Jun 28;23:235. doi: 10.1186/s13054-019-2482-x (PMC6599265; doi:10.1186/s13054-019-2482-x)
Supplement: Supplementary file 1 — Methods, STROBE statement, and supplemental tables. (DOCX 127 kb) [file 13054_2019_2482_MOESM1_ESM.docx]

**Additional File 1: Methods, STROBE statement, and supplemental tables**

**Detailed Methods:**

*Sepsis cohort study design, setting, and participants*

We used the Molecular Epidemiology of Severe Sepsis in the Intensive care unit (MESSI) cohort, an ongoing prospective cohort study of patients with sepsis and septic shock admitted to the medical intensive care unit (MICU) of the Hospital of the University of Pennsylvania (1-4). We screened all MICU admissions for patients meeting the American College of Chest Physicians/Society of Critical Care Medicine consensus criteria for severe sepsis or septic shock (5, 6). Patients were excluded if the primary reason for ICU admission was unrelated to sepsis, the patient’s goals of care included do not intubate at the time of enrollment, or if they were admitted from a long-term care facility. For the purposes of this study, we included MESSI patients admitted to the ICU from the emergency department between May 2012 and October 2014 who had plasma samples available from admission and 48 hours later (see *Plasma collection*, below). These patients did not overlap with MESSI enrollees (n=37) in whom we previously reported an association of plasma RIPK3 and blood transfusions (7). The Institutional Review Board (IRB) of the University of Pennsylvania approved the study with a waiver of timely informed consent allowing for plasma collection prior to obtaining consent. Informed consent was then obtained from subjects or their proxies.

*Trauma cohort study design, setting, and participants*

We used an ongoing prospective cohort study of critically ill trauma patients (the PEnn TRauma Organ dysfunction Study (PETROS)) admitted through the emergency department to the Penn Level I Trauma Center ICU between 2012 and 2015. All Trauma ICU patients were screened for the inclusion criteria of injury severity score (ISS) ≥16, age≥14, and admission within 24 hours of trauma. Patients were excluded for death or discharge within 24 hours of ICU admission and for isolated severe head injury defined as abbreviated injury scale (AIS) score ≥3 for head/neck and ≤1 for all other regions. Early death, which frequently occurred within several hours of presentation, was used as an exclusion criterion since it represents a competing risk with organ dysfunction—patients dying so quickly often do not have time to manifest signs of lung, kidney, or other organ injury. From this cohort, patients with plasma available at both presentation and approximately 48 hours later (see *Plasma collection*, below) were used for the primary analysis. Of note, 80 of the patients included had plasma RIPK3 levels previously reported in a study focused on AKI and blood transfusions (8). This study was approved by the Institutional Review Boards (IRBs) of both the University of Pennsylvania and the Department of Defense (DoD) since the study received financial support from a DoD award. The University of Pennsylvania IRB granted a waiver of informed consent because the research was deemed to be of minimal risk and because informed consent was deemed to make the project impracticable.

*Data collection*

Demographic, medical history, laboratory, microbiologic, physiologic, and transfusion data were collected on each patient during the first 6 days after presentation by review of the medical record by trained research personnel utilizing REDCap-based electronic case report forms (9). For sepsis patients, primary and secondary infection sources were determined by investigator consensus after review of the medical record. For trauma patients, mechanism and severity were determined by review of clinical notes and radiology reports over the first 24 hours after presentation (10).

*Outcome characterizations*

We defined ARDS over the first 6 hospital days according to the Berlin Definition (11). We collected all arterial blood gas data to calculate PaO_2_/FiO_2_. Trained physician investigators (MGSS, JPR, NJM) blinded to clinical and laboratory data evaluated all chest radiographs during the first 6 days, assessing each as “positive,” “negative,” or “equivocal,” as previously described (12). ARDS was defined as having a “positive” chest radiograph and PaO_2_/FiO_2_≤300 within 24 hours of each other. We employed the Berlin definition categories of mild, moderate, and severe based on worst PaO_2_/FiO_2_ while intubated and meeting chest radiograph criteria.

AKI was defined by Acute Kidney Injury Network (AKIN) consensus creatinine and renal replacement therapy (RRT) consensus criteria through the first 6 days (3, 13). For those diagnosed with AKI we utilized creatinine and RRT data over the subsequent 7 days for AKI staging. The AKIN creatinine criteria classify AKI as a 0.3mg/dl or 50% increase over a 48-hour time window, constituting one component of the related Kidney Disease Improving Global Outcomes (KDIGO) creatinine criteria (14). We did not incorporate the second KDIGO creatinine component, a 50% rise from pre-hospital baseline creatinine, due to lack of availability in a large percentage of patients, particularly those in the trauma cohort. Urine output criteria for AKI were not used given the high variability in urinary catheter use and data completeness over the first 6 days. Further, our study was focused on a potential pathophysiologic relationship between RIPK3 and organ dysfunction, and urine output AKI criteria are thought to be less specific than creatinine and RRT criteria for renal injury (13). Chronic dialysis patients were excluded from AKI analyses.

Mortality was determined at 30 days after admission through review of the medical record. For patients transferred to inpatient hospice within 30 days, mortality was assessed at the time of discharge from hospice.

*Plasma collection*

We utilized blood samples drawn for clinical purposes at presentation to the emergency department and approximately 48 hours after presentation. According to clinical protocol, these samples were drawn into citrated vacutainers and centrifuged at 3,000 g within 30 minutes to separate plasma for testing of coagulation parameters. Residual sample was immediately refrigerated at 4°C. Study personnel obtained these samples within 12 to 48 hours and froze plasma aliquots at -80°C. We have previously demonstrated the feasibility of using blood samples collected in this fashion for studies of plasma biomarkers, including RIPK3, in critical illness populations (1, 2, 8, 15).

*Plasma RIPK3 measurement*

We used a commercially available enzyme-linked immunosorbent assay (CUSABIO, Wuhan, China) to measure plasma RIPK3 concentrations (7, 8). Performing personnel were blind to clinical data including ARDS, AKI, and mortality status. Plasma concentrations below the limit of detection (15.6 pg/ml) were set to 15.6 pg/ml for statistical analysis.

*Statistical analysis*

In primary analyses, by cohort (sepsis, trauma) we tested the unadjusted associations of patient characteristics, including plasma RIPK3 levels, with ARDS using the t-test, Wilcoxon rank-sum test, χ^2^ test, or Fisher’s exact test, as appropriate. We used separate multivariable logistic regression models to test the association of ΔRIPK3 with ARDS adjusted for confounders, assessing model fit with the Hosmer-Lemeshow goodness-of-fit test (16). To avoid overfitting, we limited explanatory variables in each model to approximately one for every ten outcomes (17). We pre-specified these variables by cohort based on previously described associations with ARDS or RIPK3 (7, 8, 18):

Sepsis: ΔRIPK3, age, red blood cell (RBC) transfusions, sepsis source, shock

Trauma: ΔRIPK3, RBC transfusions, trauma mechanism, injury severity score

We did not utilize APACHE II as an explanatory variable for ARDS since the oxygenation component of APACHE II is also a component of the Berlin ARDS definition. We utilized post-estimation marginal analysis to determine adjusted risk of ARDS across a range of plasma ΔRIPK3 levels (19).

We repeated the primary ΔRIPK3-ARDS analyses stratified by several patient characteristics and tested for interaction using likelihood ratio tests. For sepsis, strata included shock versus no shock and pulmonary versus non-pulmonary source of infection. For trauma, we stratified by blunt versus penetrating injury mechanism.

We conducted several secondary analyses. First, we determined the associations of ΔRIPK3 with AKI and mortality in each cohort using similar methods to those in the primary analyses. Similar to the multivariable models for ARDS, we pre-specified explanatory variables by cohort based on previously described associations with RIPK3, AKI, or mortality in order to avoid model overfitting (3, 8, 20, 21):

Sepsis-AKI: ΔRIPK3, age, RBC transfusions, chronic kidney disease, diabetes mellitus, shock at presentation

Trauma-AKI: ΔRIPK3, RBC transfusions, trauma mechanism, abdominal injury severity, shock prior to ICU admission

Sepsis-mortality: ΔRIPK3, age, RBC transfusions, immunocompromised, shock at presentation

We did not create a multivariable model for mortality in the trauma population given the limited number who died (n=17). We also determined differences in ΔRIPK3 by organ dysfunction categories: ARDS alone, AKI alone, or both, using Wilcoxon rank-sum testing. We used multivariable logistic regression to determine if the association of ΔRIPK3 with ARDS was independent of AKI, and vice versa. Finally, we tested the associations of baseline characteristics with RIPK3 levels using Wilcoxon rank-sum, Kruskal-Wallis tests, or Spearman’s rank correlation, as appropriate.

Missing covariate data were uncommon, were treated with a complete case analysis approach (22), and are noted in tables when applicable. We estimated sample size separately for sepsis and trauma cohorts, anticipating that ARDS rates would each be approximately 40% in sepsis and 20% in trauma (1, 3, 23). At an α of 0.05, a sepsis sample size of 120 and trauma sample size of 180 would give 91% and 89% power, respectively, to detect an average difference of 131 pg/ml between ARDS cases and non-cases. We considered 131 pg/ml potentially clinically relevant since it was the difference in plasma RIPK3 between patients with and without AKI in our prior pilot study (8). We used Stata/IC 13.1 (StataCorp, College Station, TX) and considered a two-tailed p<0.05 significant for all analyses.

***Experimental animal studies***

**C57Bl/6 animals were purchased from the Charles River Laboratories Inc. All experimental procedures were performed on 8-12 week-old female mice, 20-25g in weight. Animal studies were conducted in accordance with the Institutional Animal Care and Use Committee at the University of Pennsylvania.**

**Mice were injected via tail vein with 10 mg/kg LPS (List Labs) as well as 10 mg/kg of the pan-caspase inhibitor ZVAD-FMK (BD Biosciences) in order to inhibit apoptosis and sensitize cells to necroptosis as previously described (24-28). Four hours following LPS-ZVAD administration, mice were sacrificed with intraperitoneal injections of ketamine/xylazine (80/10 mg/kg). Whole blood was obtained via cardiac puncture and spun for five minutes at 5000rpm to isolate plasma. Whole lungs were frozen for western blots analysis.**

Lungs were homogenized in cell lysis buffer, sonicated, and centrifuged for 10 min at 16,000 g. Proteins were resolved by SDS-PAGE under reducing conditions. Immunoblotting was performed using the following antibodies: rabbit α-RIPK3 (Prosci), rabbit α-MLKL (Abcam), rabbit α-phosphorylated MLKL (Abcam), and rabbit α-*B* actin (Licor). Bands were visualized using Licor Odyssey and densitometry was quantified using ImageStudio Lite software. We used Sigmaplot Software (Systat Software Inc.) to construct graphs.

Murine plasma RIPK3 was measured using RIPK3 ELISA kit (Cusabio). Lung tissue RIPK3 was normalized to β-actin. We determined differences in plasma and tissue RIPK3 between control and LPS groups using the Wilcoxon rank-sum test and tested the Spearman rank correlation between plasma and tissue RIPK3 (Stata/IC 13.1 (StataCorp, College Station, TX)).

**References:**

1. Palakshappa JA, Anderson BJ, Reilly JP, Shashaty MG, Ueno R, Wu Q, et al. Low Plasma Levels of Adiponectin Do Not Explain Acute Respiratory Distress Syndrome Risk: a Prospective Cohort Study of Patients with Severe Sepsis. Crit Care. 2016;20(1):71.

2. Reilly JP, Anderson BJ, Hudock KM, Dunn TG, Kazi A, Tommasini A, et al. Neutropenic sepsis is associated with distinct clinical and biological characteristics: a cohort study of severe sepsis. Crit Care. 2016;20(1):222.

3. Reilly JP, Anderson BJ, Mangalmurti NS, Nguyen TD, Holena DN, Wu Q, et al. The ABO Histo-Blood Group and AKI in Critically Ill Patients with Trauma or Sepsis. Clin J Am Soc Nephrol. 2015;10(11):1911-20.

4. Reilly JP, Meyer NJ, Shashaty MG, Feng R, Lanken PN, Gallop R, et al. ABO blood type A is associated with increased risk of ARDS in whites following both major trauma and severe sepsis. Chest. 2014;145(4):753-61.

5. Bone RC, Balk RA, Cerra FB, Dellinger RP, Fein AM, Knaus WA, et al. Definitions for sepsis and organ failure and guidelines for the use of innovative therapies in sepsis. The ACCP/SCCM Consensus Conference Committee. American College of Chest Physicians/Society of Critical Care Medicine. Chest. 1992;101(6):1644-55.

6. Levy MM, Fink MP, Marshall JC, Abraham E, Angus D, Cook D, et al. 2001 SCCM/ESICM/ACCP/ATS/SIS International Sepsis Definitions Conference. Critical care medicine. 2003;31(4):1250-6.

7. Qing DY, Conegliano D, Shashaty MG, Seo J, Reilly JP, Worthen GS, et al. Red blood cells induce necroptosis of lung endothelial cells and increase susceptibility to lung inflammation. Am J Respir Crit Care Med. 2014;190(11):1243-54.

8. Shashaty MG, Reilly JP, Sims CA, Holena DN, Qing D, Forker CM, et al. Plasma Levels of Receptor Interacting Protein Kinase-3 (RIP3), an Essential Mediator of Necroptosis, are Associated with Acute Kidney Injury in Critically Ill Trauma Patients. Shock. 2016.

9. Harris PA, Taylor R, Thielke R, Payne J, Gonzalez N, Conde JG. Research electronic data capture (REDCap)--a metadata-driven methodology and workflow process for providing translational research informatics support. Journal of biomedical informatics. 2009;42(2):377-81.

10. Baker SP, O'Neill B, Haddon Jr W, Long WB. The injury severity score: a method for describing patients with multiple injuries and evaluating emergency care. Journal of Trauma. 1974;14(3):187-96.

11. Force TADT. Acute respiratory distress syndrome: The berlin definition. JAMA. 2012;307(23):2526-33.

12. Shah CV, Lanken PN, Localio AR, Gallop R, Bellamy S, Ma SF, et al. An alternative method of acute lung injury classification for use in observational studies. Chest. 2010;138(5):1054-61.

13. Mehta RL, Kellum JA, Shah SV, Molitoris BA, Ronco C, Warnock DG, et al. Acute Kidney Injury Network: report of an initiative to improve outcomes in acute kidney injury. Critical care (London, England). 2007;11(2).

14. Kellum JA, Lameire N, Aspelin P, Barsoum RS, Burdmann EA, Goldstein SL, et al. Kidney disease: Improving global outcomes (KDIGO) acute kidney injury work group. KDIGO clinical practice guideline for acute kidney injury. Kidney International Supplements. 2012;2(1):1-138.

15. Anderson BJ, Reilly JP, Shashaty MG, Palakshappa JA, Wysoczanski A, Dunn TG, et al. Admission plasma levels of the neuronal injury marker neuron-specific enolase are associated with mortality and delirium in sepsis. J Crit Care. 2016;36:18-23.

16. Hosmer Jr DW, Lemeshow S, Sturdivant RX. Applied logistic regression: John Wiley & Sons; 2013.

17. Peduzzi P, Concato J, Kemper E, Holford TR, Feinstein AR. A simulation study of the number of events per variable in logistic regression analysis. J Clin Epidemiol. 1996;49(12):1373-9.

18. Watkins TR, Nathens AB, Cooke CR, Psaty BM, Maier RV, Cuschieri J, et al. Acute respiratory distress syndrome after trauma: development and validation of a predictive model. Crit Care Med. 2012;40(8):2295-303.

19. Graubard BI, Korn EL. Predictive margins with survey data. Biometrics. 1999;55(2):652-9.

20. Shashaty MG, Meyer NJ, Localio AR, Gallop R, Bellamy SL, Holena DN, et al. African American race, obesity, and blood product transfusion are risk factors for acute kidney injury in critically ill trauma patients. J Crit Care. 2012;27(5):496-504.

21. Plataki M, Kashani K, Cabello-Garza J, Maldonado F, Kashyap R, Kor DJ, et al. Predictors of acute kidney injury in septic shock patients: an observational cohort study. Clin J Am Soc Nephrol. 2011;6(7):1744-51.

22. Pigott TD. A review of methods for missing data. Educational research and evaluation. 2001;7(4):353-83.

23. Reilly JP, Bellamy S, Shashaty MG, Gallop R, Meyer NJ, Lanken PN, et al. Heterogeneous phenotypes of acute respiratory distress syndrome after major trauma. Ann Am Thorac Soc. 2014;11(5):728-36.

24. Wu YT, Tan HL, Huang Q, Sun XJ, Zhu X, Shen HM. zVAD-induced necroptosis in L929 cells depends on autocrine production of TNFalpha mediated by the PKC-MAPKs-AP-1 pathway. Cell Death Differ. 2011;18(1):26-37.

25. Zelic M, Roderick JE, O'Donnell JA, Lehman J, Lim SE, Janardhan HP, et al. RIP kinase 1-dependent endothelial necroptosis underlies systemic inflammatory response syndrome. J Clin Invest. 2018;128(5):2064-75.

26. Duprez L, Takahashi N, Van Hauwermeiren F, Vandendriessche B, Goossens V, Vanden Berghe T, et al. RIP kinase-dependent necrosis drives lethal systemic inflammatory response syndrome. Immunity. 2011;35(6):908-18.

27. Kaiser WJ, Sridharan H, Huang C, Mandal P, Upton JW, Gough PJ, et al. Toll-like receptor 3-mediated necrosis via TRIF, RIP3, and MLKL. J Biol Chem. 2013;288(43):31268-79.

28. Moreno-Gonzalez G, Vandenabeele P, Krysko DV. Necroptosis: A Novel Cell Death Modality and Its Potential Relevance for Critical Care Medicine. American Journal of Respiratory and Critical Care Medicine. 2016;194(4):415-28.

**STROBE Statement:**

Checklist of items that should be included in reports of ***cohort studies***

|  | Item No | Recommendation |
| --- | --- | --- |
| **Title and abstract** | 1 | (*a*) Indicate the study’s design with a commonly used term in the title or the abstract  **SEE ABSTRACT.** |
|  |  | (*b*) Provide in the abstract an informative and balanced summary of what was done and what was found  **SEE ABSTRACT.** |
| Introduction | | |
| Background/rationale | 2 | Explain the scientific background and rationale for the investigation being reported  **SEE INTRODUCTION PARAGRAPHS 2 AND 3.** |
| Objectives | 3 | State specific objectives, including any prespecified hypotheses  **SEE INTRODUCTION PARAGRAPH 4.** |
| Methods | | |
| Study design | 4 | Present key elements of study design early in the paper  **SEE “SEPSIS AND TRAUMA COHORTS” SUBHEADING IN METHODS.** |
| Setting | 5 | Describe the setting, locations, and relevant dates, including periods of recruitment, exposure, follow-up, and data collection  **SEE “SEPSIS AND TRAUMA COHORTS” SUBHEADING IN METHODS AS WELL AS “STUDY DESIGN, SETTING, AND PARTICIPANTS” FOR BOTH SEPSIS AND TRAUMA COHORTS IN ADDITIONAL FILE 1.** |
| Participants | 6 | (*a*) Give the eligibility criteria, and the sources and methods of selection of participants. Describe methods of follow-up  **SEE “SEPSIS AND TRAUMA COHORTS” SUBHEADING IN METHODS AS WELL AS “STUDY DESIGN, SETTING, AND PARTICIPANTS” FOR BOTH SEPSIS AND TRAUMA COHORTS IN ADDITIONAL FILE 1.** |
|  |  | (*b*) For matched studies, give matching criteria and number of exposed and unexposed  **NOT APPLICABLE.** |
| Variables | 7 | Clearly define all outcomes, exposures, predictors, potential confounders, and effect modifiers. Give diagnostic criteria, if applicable  **SEE “SEPSIS AND TRAUMA COHORTS,” “DATA COLLECTION AND OUTCOMES,” “PLASMA COLLECTION AND RIPK3 MEASUREMENT,” AND “STATISTICAL ANALYSIS” SUBHEADINGS IN METHODS AS WELL AS “STUDY DESIGN, SETTING, AND PARTICIPANTS” FOR BOTH SEPSIS AND TRAUMA COHORTS, “DATA COLLECTION,” “OUTCOME CHARACTERIZATIONS,” “PLASMA RIPK3 MEASUREMENT,” AND “STATISTICAL ANALYSIS” IN ADDITIONAL FILE 1.** |
| Data sources/ measurement | 8* | For each variable of interest, give sources of data and details of methods of assessment (measurement). Describe comparability of assessment methods if there is more than one group  **SEE “DATA COLLECTION AND OUTCOMES” AND “PLASMA COLLECTION AND RIPK3 MEASUREMENT” SUBHEADINGS IN METHODS AS WELL AS “DATA COLLECTION,” “OUTCOME CHARACTERIZATIONS,” AND “PLASMA RIPK3 MEASUREMENT” IN ADDITIONAL FILE 1.** |
| Bias | 9 | Describe any efforts to address potential sources of bias  **SEE “STATISTICAL ANALYSIS” SUBHEADING IN METHODS AND “STATISTICAL ANALYSIS” IN ADDITIONAL FILE 1.** |
| Study size | 10 | Explain how the study size was arrived at  **SEE “STATISTICAL ANALYSIS” IN ADDITIONAL FILE 1, 6^TH^ PARAGRAPH, “WE ESTIMATED SAMPLE SIZE…”** |
| Quantitative variables | 11 | Explain how quantitative variables were handled in the analyses. If applicable, describe which groupings were chosen and why  **SEE “PLASMA COLLECTION AND RIPK3 MEASUREMENT” SUBHEADING IN METHODS, AND “PLASMA RIPK3 MEASUREMENT” IN ADDITIONAL FILE 1.** |
| Statistical methods | 12 | (*a*) Describe all statistical methods, including those used to control for confounding  **SEE “STATISTICAL ANALYSIS” IN METHODS AND ADDITIONAL FILE 1** |
|  |  | (*b*) Describe any methods used to examine subgroups and interactions  **SEE “STATISTICAL ANALYSIS” IN METHODS, 2^ND^ PARAGRAPH, AND “STATISTICAL ANALYSIS” IN ADDITIONAL FILE 1 3^RD^ PARAGRAPH** |
|  |  | (*c*) Explain how missing data were addressed  **SEE “STATISTICAL ANALYSIS” IN ADDITIONAL FILE 1, 6^TH^ PARAGRAPH “MISSING COVARIATE DATA WERE UNCOMMON…”** |
|  |  | (*d*) If applicable, explain how loss to follow-up was addressed  **NOT APPLICABLE.** |
|  |  | (*e*) Describe any sensitivity analyses  **SEE “STATISTICAL ANALYSIS” IN METHODS, 2^ND^ PARAGRAPH, AND “STATISTICAL ANALYSIS” IN ADDITIONAL FILE 1, 4^TH^ AND 5^TH^ PARAGRAPHS** |
| Results | | |
| Participants | 13* | (a) Report numbers of individuals at each stage of study—eg numbers potentially eligible, examined for eligibility, confirmed eligible, included in the study, completing follow-up, and analysed  **SEE RESULTS PARAGRAPHS 1 AND 2, SUPPLEMENTAL TABLE 1 (IN ADDITIONAL FILE 1).** |
|  |  | (b) Give reasons for non-participation at each stage  **SEE RESULTS PARAGRAPH 1 (PATIENTS EXCLUDED IF PLASMA NOT AVAILABLE AT BOTH TIME POINTS).** |
|  |  | (c) Consider use of a flow diagram  **OMITTED—STRAIGHTFORWARD AND DESCRIBED IN PARAGRAPH 1, SUPPLEMENTAL TABLE 1 (IN ADDITIONAL FILE 1).** |
| Descriptive data | 14* | (a) Give characteristics of study participants (eg demographic, clinical, social) and information on exposures and potential confounders  **SEE “MESSI COHORT” AND “PETROS COHORT” IN RESULTS, TABLE 1.** |
|  |  | (b) Indicate number of participants with missing data for each variable of interest  **TABLE 1 AND SUPPLEMENTAL TABLE 4 (IN ADDITIONAL FILE 1) CAPTIONS.** |
|  |  | (c) Summarise follow-up time (eg, average and total amount)  **DESCRIBED IN METHODS: 6 DAYS FOLLOW-UP FOR ARDS AND AKI. MORTALITY ASSESSED AT 30 DAYS.** |
| Outcome data | 15* | Report numbers of outcome events or summary measures over time  **SEE “MESSI COHORT” AND “PETROS COHORT” IN RESULTS.** |
| Main results | 16 | (*a*) Give unadjusted estimates and, if applicable, confounder-adjusted estimates and their precision (eg, 95% confidence interval). Make clear which confounders were adjusted for and why they were included  **SEE TABLES 1-4, SUPPLEMENTAL TABLES, AND RESULTS 5^TH^ PARAGRAPH. ALSO SEE METHODS (STATISTICAL ANALYSIS) FOR CONFOUNDER SELECTION STRATEGY.** |
|  |  | (*b*) Report category boundaries when continuous variables were categorized  **SEE TABLE 3 AND SUPPLEMENTAL TABLE 3 (IN ADDITIONAL FILE 1).** |
|  |  | (*c*) If relevant, consider translating estimates of relative risk into absolute risk for a meaningful time period  **MULTIVARIABLE ASSOCIATIONS WITH ARDS PRESENTED AS ODDS RATIOS (TABLE 2).** |
| Other analyses | 17 | Report other analyses done—eg analyses of subgroups and interactions, and sensitivity analyses  **SEE RESULTS 1^ST^ AND 5^TH^ PARAGRAPHS, SUPPLEMENTAL TABLES.** |
| Discussion | | |
| Key results | 18 | Summarise key results with reference to study objectives |
| Limitations | 19 | Discuss limitations of the study, taking into account sources of potential bias or imprecision. Discuss both direction and magnitude of any potential bias  **SEE 5^TH^ PARAGRAPH, “OUR STUDY HAS SEVERAL LIMITATIONS…”** |
| Interpretation | 20 | Give a cautious overall interpretation of results considering objectives, limitations, multiplicity of analyses, results from similar studies, and other relevant evidence  **SEE DISCUSSION (ENTIRETY).** |
| Generalisability | 21 | Discuss the generalisability (external validity) of the study results  **SEE DISCUSSION 5^TH^ PARAGRAPH.** |
| Other information | | |
| Funding | 22 | Give the source of funding and the role of the funders for the present study and, if applicable, for the original study on which the present article is based  **SEE FUNDING STATEMENT AT BOTTOM OF TITLE SECTION.** |

*Give information separately for exposed and unexposed groups.

**Note:** An Explanation and Elaboration article discusses each checklist item and gives methodological background and published examples of transparent reporting. The STROBE checklist is best used in conjunction with this article (freely available on the Web sites of PLoS Medicine at http://www.plosmedicine.org/, Annals of Internal Medicine at http://www.annals.org/, and Epidemiology at http://www.epidem.com/). Information on the STROBE Initiative is available at http://www.strobe-statement.org.

**Supplemental Table 1. Multivariable logistic regression models of the ΔRIPK3-AKI association adjusted for pre-specified confounders.**

1. **MESSI Cohort.**

| **Covariate** | **Odds ratio (95% CI)** | ***p*** |
| --- | --- | --- |
| ΔRIPK3 (per ½ SD) | 1.32 (1.06-1.65) | 0.013 |
| Age | 1.00 (0.97-1.03) | 0.877 |
| RBCs transfused, presentation day (per unit) | 0.81 (0.47-1.40) | 0.448 |
| Chronic kidney disease | 2.03 (0.52-7.86) | 0.305 |
| Diabetes mellitus | 2.31 (0.96-5.53) | 0.060 |
| Shock at presentation*^a^* | 0.63 (0.24-1.67) | 0.355 |

1. **PETROS Cohort.**

| **Covariate** | **Odds ratio (95% CI)** | ***p*** |
| --- | --- | --- |
| ΔRIPK3 (per ½ SD) | 1.39 (1.10-1.76) | 0.006 |
| RBCs transfused, first 6h (per unit) | 1.01 (0.89-1.14) | 0.935 |
| Blunt trauma mechanism | 0.51 (0.22-1.20) | 0.121 |
| AIS score for abdomen (per point) | 1.29 (1.02-1.62) | 0.033 |
| Shock prior to ICU admission*^a^* | 2.11 (0.94-4.70) | 0.069 |

For **A.** and **B.**: ΔRIPK3 remains significantly associated with AKI after adjustment for pre-specified confounders. The odds ratio corresponds to the adjusted association of each covariate with AKI. Note: patients with end-stage renal disease were excluded for this analysis (MESSI Cohort, n=9; PETROS Cohort, n=1;). *^a^*Shock defined as need for vasopressors or mean arterial pressure <65 mmHg (MESSI) or systolic arterial pressure <90 mmHg (PETROS). Definition of abbreviations: AKI: acute kidney injury; RIPK3: receptor interacting protein kinase-3; SD: standard deviation; RBCs: red blood cells; AIS: abbreviated injury scale.

**Supplemental Table 2. Impact of including both ARDS and AKI in models of the associations of ΔRIPK3 with organ dysfunction.**

1. **ARDS as outcome**

| **Model** | **OR (95% CI) for ΔRIPK3-ARDS** | ***p*** |
| --- | --- | --- |
| MESSI |  |  |
| Primary | 1.30 (1.03-1.63) | 0.027 |
| Primary + AKI | 1.25 (0.97-1.60) | 0.081 |
| PETROS |  |  |
| Primary | 1.83 (1.35-2.48) | <0.001 |
| Primary + AKI | 1.78 (1.31-2.42) | <0.001 |

1. **AKI as outcome**

| **Model** | **OR (95% CI) for ΔRIPK3-AKI** | ***p*** |
| --- | --- | --- |
| MESSI |  |  |
| Primary | 1.32 (1.06-1.65) | 0.013 |
| Primary + ARDS | 1.26 (1.01-1.58) | 0.044 |
| PETROS |  |  |
| Primary | 1.39 (1.10-1.76) | 0.006 |
| Primary + ARDS | 1.35 (1.05-1.73) | 0.019 |

Primary multivariable models for ARDS are those shown in Table 3. Primary + AKI models also include AKI as an explanatory variable for ARDS. Primary multivariable models for AKI are those shown in Supplemental Table 1. Odds ratios (OR) are per ½ standard deviation of ΔRIPK3 concentration. Primary + ARDS models also include ARDS as an explanatory variable for AKI. Definition of abbreviations: ARDS: acute respiratory distress syndrome; AKI: acute kidney injury; RIPK3: receptor interacting protein kinase-3.

**Supplemental Table 3. Multivariable logistic regression model of the ΔRIPK3-mortality association in the MESSI Cohort adjusted for pre-specified confounders.**

| **Covariate** | **Odds ratio (95% CI)** | ***p*** |
| --- | --- | --- |
| ΔRIPK3 (per ½ SD) | 1.27 (1.03-1.57) | 0.028 |
| Age | 1.03 (1.00-1.05) | 0.049 |
| RBCs transfused, presentation day (per unit) | 1.24 (0.88-1.74) | 0.223 |
| Immunocompromise*^a^* | 1.67 (0.77-3.68) | 0.203 |
| Shock at presentation*^b^* | 0.98 (0.39-2.45) | 0.459 |

In the MESSI Cohort, ΔRIPK3 remains significantly associated with 30-day mortality after adjustment for pre-specified confounders. The odds ratio corresponds to the adjusted association of each covariate with mortality. *^a^*Defined as patients with a malignancy undergoing cytotoxic chemotherapy or patients with the acquired immune deficiency syndrome. *^b^*Shock defined as need for vasopressors or mean arterial pressure <65 mmHg. Definition of abbreviations: ΔRIPK3=change in plasma receptor interacting protein kinase-3 levels from presentation to 48 hours; SD=standard deviation; RBCs=red blood cells. Note: No multivariable model was constructed for ΔRIPK3-mortality in the PETROS cohort since the limited number of deaths (n=17) would have resulted in an overfit model.

**Supplemental Table 4.** **Unadjusted associations of baseline characteristics with the change in receptor interacting protein kinase-3 plasma concentrations from presentation to 48 hours (ΔRIPK3).**

|  | **MESSI Cohort** | | **PETROS Cohort** | |
| --- | --- | --- | --- | --- |
|  | **ΔRIPK3 (pg/ml) presentation to 48h** | ***p*** | **ΔRIPK3 (pg/ml) presentation to 48h** | ***p*** |
| Demographics |  |  |  |  |
| Age, years*^a^*  Tertile 1  Tertile 2  Tertile 3 | 11 (-61, 113)  0 (-60, 72)  24 (-14, 91) | 0.437 | 87 (9-188)  96 (0-186)  38 (0-133) | 0.033 |
| Sex  Female  Male | 12 (-20, 72)  20 (-60, 151) | 0.917 | 31 (0-125)  68 (3-181) | 0.088 |
| Race*^b^*  White  African American  Other*^c^* | 0 (-51, 100)  19 (-36, 262)  19 (0, 64) | 0.917 | 30 (0-131)  90 (27-178)  118 (19-196) | 0.029*^h^* |
| Body mass index*^d^* (kg/m^2^)  Underweight  Normal weight  Overweight  Obese | 52 (-16, 98)  0 (-68, 32)  10 (-57, 158)  45 (-12, 262) | 0.073 | 56 (0-220)  70 (0-156)  61 (3-175)  47 (0-139) | 0.443 |
| Medical History |  |  |  |  |
| Hypertension*^b^*  Present  Absent | 21 (-14, 100)  0 (-65, 100) | 0.168 | 40 (0-146)  73 (0-151) | 0.166 |
| Diabetes mellitus*^b^*  Present  Absent | 23 (-7, 172)  0 (-59, 82) | 0.189 | 24 (-29-70)  66 (0-154) | 0.102 |
| Congestive heart failure*^b^*  Present  Absent | 0 (-14, 185)  18 (-51, 100) | 0.812 | 70 (0-186)  61 (0-151) | 0.776 |
| Chronic kidney disease*^b^*  Present  Absent | 0 (-56, 164)  19 (-38, 100) | 0.789 | 24 (12-27)  64 (0-152) | 0.620 |
| Chronic alcohol abuse*^b^*  Present  Absent | 0 (-209, 603)  6 (-51, 76) | 0.962 | 29 (0-128)  62 (0-151) | 0.400 |
| Smoking history*^b^*  Never  Former  Current | 17 (-55, 113)  31 (-34, 151)  -2 (-60, 50) | 0.538 | 48 (0-122)  54 (0-196)  61 (0-175) | 0.230*^i^* |
| Acute injury and illness |  |  |  |  |
| Blunt trauma mechanism  Yes  No | N/A |  | 43 (0-139)  128 (56-295) | <0.001 |
| Injury Severity Score*^a^*  Tertile 1  Tertile 2  Tertile 3 | N/A |  | 61 (0-129)  54 (0-171)  96 (0-179) | 0.986 |
| Operation prior to ICU admission  Yes  No | N/A |  | 116 (43-231)  37 (0-129) | <0.001 |
| APACHE II*^a^*  Tertile 1  Tertile 2  Tertile 3 | -7 (-61, 19)  16 (-60, 65)  117 (18, 409) | <0.001 | 18 (0-109)  57 (0-150)  132 (43-225) | <0.001 |
| Shock prior to ICU admission*^be^*  Yes  No | 19 (-28, 100)  0 (-60, 106) | 0.613 | 109 (27-218)  43 (0-128) | 0.004 |
| Crystalloid, liters*^abf^*  Tertile 1  Tertile 2  Tertile 3 | 19 (-43, 71)  11 (-51, 82)  23 (-59, 151) | 0.388 | 15 (0-122)  66 (0-154)  116 (47-240) | <0.001 |
| Pulmonary source  Yes  No | 1 (-34, 58)  22 (-51, 120) | 0.475 | N/A |  |
| Transfusions |  |  |  |  |
| RBC units, day 0+1*^g^*  None  1-2  3-5  ≥6 | 0 (-34, 64)  66 (-60, 200)  59 (-2, 120)  - | 0.131 | 12 (0-84)  47 (0-128)  90 (27-231)  181 (111-328) | <0.001 |
| Plasma units, day 0+1*^g^*  None  1-4  ≥5 | 19 (-38, 94)  -20 (-65, 262)  19 (-2, 185) | 0.760 | 30 (0-90)  124 (46-222)  186 (111-359) | <0.001 |
| Platelet doses, day 0+1*^g^*  None  1  ≥5 | 0 (-51, 64)  110 (-5, 531)  687 (185, 1190) | 0.017 | 43 (0-118)  107 (5-226)  164 (70-290) | <0.001 |
| 30-day mortality  Yes  No | 29 (-16, 290)  0 (-60, 72) | 0.049 | 152 (43-231)  56 (0-146) | 0.022 |

Plasma receptor interacting protein kinase-3 (RIPK3) concentrations are shown as median (interquartile range). All p-values are from comparisons using Wilcoxon rank-sum test (categorical characteristics) or Spearman’s correlation (continuous characteristics) unless otherwise specified. *^a^*Tertiles of continuous covariates are from lowest to highest values. *^b^*Missing data as follows: for MESSI (sepsis) cohort, chronic alcohol abuse (n=19); smoking history (18); crystalloid (2); for PETROS (trauma) cohort, race (n=4), hypertension (4), diabetes (3), coronary heart disease (3), congestive heart failure (2), chronic kidney disease (1), chronic alcohol abuse (5), smoking history (17), shock prior to ICU admission (1), crystalloid (4). *^c^*For PETROS cohort, Asian (n=9) and North American Indian/Alaskan Native (n=2); for MESSI cohort, Asian (n=1) and Unknown (n=5). *^d^*Body mass index categories according to World Health Organization. *^e^*Shock defined as need for vasopressors or mean arterial pressure <65 mmHg (MESSI) or systolic arterial pressure <90 mmHg (PETROS). *^f^*Administered during the first 24 hours after ED presentation (MESSI) or prior to ICU arrival (PETROS). *^g^*The calendar day of and the day after presentation. *^h^*Kruskal-Wallis test. *^i^*Cuzick’s non-parametric test of trend across smoking categories. Definition of abbreviations: APACHE: Acute Physiology And Chronic Health Evaluation; RBC: red blood cell.

**Supplemental Table 5.** **Unadjusted associations of baseline characteristics with receptor interacting protein kinase-3 (RIPK3) plasma concentrations at presentation.**

|  | **MESSI Cohort** | | **PETROS Cohort** | |
| --- | --- | --- | --- | --- |
|  | **RIPK3 (pg/ml), presentation** | ***p*** | **RIPK3 (pg/ml), presentation** | ***p*** |
| Demographics |  |  |  |  |
| Age, years*^a^*  Tertile 1 (16-28)  Tertile 2 (29-56)  Tertile 3 (57-91) | 161 (103-450)  175 (57-390)  105 (34-266) | 0.008 | 4 (4-45)  4 (4-63)  4 (4-49) | 0.917 |
| Sex  Female  Male | 80 (35-221)  150 (52-321) | 0.070 | 4 (4-45)  4 (4-48) | 0.921 |
| Race*^b^*  Caucasian  African American  Other*^c^* | 113 (17-266)  138 (56-321)  75 (15-235) | 0.273 | 4 (4-49)  4 (4-45)  4 (4-80) | 0.567 |
| Body mass index*^d^* (kg/m^2^)  Underweight  Normal weight  Overweight  Obese | 105 (39-321)  117 (42-287)  167 (63-415)  94 (36-261) | 0.429 | 10 (4-28)  4 (4-47)  4 (4-69)  4 (4-42) | 0.670 |
| Medical History |  |  |  |  |
| Hypertension*^b^*  Present  Absent | 97 (33-286)  133 (67-307) | 0.158 | 4 (4-50)  4 (4-47) | 0.575 |
| Diabetes mellitus*^b^*  Present  Absent | 117 (36-265)  131 (39-314) | 0.625 | 4 (4-107)  4 (4-46) | 0.459 |
| Congestive heart failure*^b^*  Present  Absent | 64 (16-157)  134 (48-307) | 0.079 | 81 (4-176)  4 (4-46) | 0.031 |
| Chronic kidney disease*^b^*  Present  Absent | 112 (27-284)  121 (43-289) | 0.592 | 4 (4-4)  4 (4-47) | 0.304 |
| Chronic alcohol abuse*^b^*  Present  Absent | 321 (265-454)  121 (36-257) | 0.005 | 4 (4-63)  4 (4-47) | 0.630 |
| Smoking history*^b^*  Never  Former  Current | 147 (55-335)  104 (18-257)  134 (36-390) | 0.577 | 4 (4-73)  4 (4-4)  4 (4-47) | 0.939 |
| Injury characteristics |  |  |  |  |
| Blunt trauma mechanism  Yes  No | N/A |  | 4 (4-63)  4 (4-14) | 0.023 |
| Injury Severity Score*^a^*  Tertile 1 (16-21)  Tertile 2 (22-29)  Tertile 3 (30-66) | N/A |  | 4 (4-44)  4 (4-37)  25 (4-126) | 0.054 |
| Operation prior to ICU admission  Yes  No | N/A |  | 4 (4-46)  4 (4-49) | 0.575 |
| APACHE II*^a^*  Tertile 1  Tertile 2  Tertile 3 | 69 (34-187)  133 (48-286)  223 (65-381) | 0.034 | 4 (4-45)  4 (4-42)  4 (4-64) | 0.348 |
| Shock prior to ICU admission*^be^*  Yes  No | 117 (36-291)  120 (48-283) | 0.932 | 4 (4-46)  4 (4-50) | 0.180 |
| Crystalloid, liters*^abf^*  Tertile 1  Tertile 2  Tertile 3 | 60 (15-134)  144 (58-283)  175 (65-421) | 0.001 | 8 (4-49)  4 (4-46)  4 (4-58) | 0.562 |
| Transfusions |  |  |  |  |
| RBC units, day 0+1*^g^*  None  1-2  3-5  ≥6 | 106 (34-265)  187 (58-417)  134 (117-139)  - | 0.106 | 4 (4-28)  26 (4-68)  4 (4-132)  4 (4-38) | 0.949 |
| Plasma units, day 0+1*^g^*  None  1-4  ≥5 | 113 (36-274)  261 (144-421)  117 (15-261) | 0.054 | 4 (4-49)  4 (4-55)  4 (4-41) | 0.404 |
| Platelet doses, day 0+1*^g^*  None  1-5  ≥5 | 113 (36-261)  272 (99-452)  260 (15-507) | 0.020 | 4 (4-47)  4 (4-65)  4 (4-41) | 0.624 |
| 30-day mortality  Yes  No | 150 (49-413)  110 (34-235) | 0.068 | 4 (4-81)  4 (4-47) | 0.439 |

Plasma receptor interacting protein kinase-3 (RIPK3) concentrations are shown as median (interquartile range). All p-values are from comparisons using Wilcoxon rank-sum test (categorical characteristics) or Spearman’s correlation (continuous characteristics) unless otherwise specified. *^a^*Tertiles of continuous covariates are from lowest to highest values. *^b^*Missing data as follows: for MESSI (sepsis) cohort, chronic alcohol abuse (n=19); smoking history (18); crystalloid (2); for PETROS (trauma) cohort, race (n=4), hypertension (4), diabetes (3), coronary heart disease (3), congestive heart failure (2), chronic kidney disease (1), chronic alcohol abuse (5), smoking history (17), shock prior to ICU admission (1), crystalloid (4). *^c^*For PETROS cohort, Asian (n=9) and North American Indian/Alaskan Native (n=2); for MESSI cohort, Asian (n=1) and Unknown (n=5). *^d^*Body mass index categories according to World Health Organization. *^e^*Shock defined as need for vasopressors or mean arterial pressure <65 mmHg (MESSI) or systolic arterial pressure <90 mmHg (PETROS). *^f^*Administered during the first 24 hours after ED presentation (MESSI) or prior to ICU arrival (PETROS). *^g^*The calendar day of and the day after presentation. *^h^*Kruskal-Wallis test. *^i^*Cuzick’s non-parametric test of trend across smoking categories. Definition of abbreviations: APACHE: Acute Physiology And Chronic Health Evaluation; RBC: red blood cell.
