# Supplementary figures and images for: Plasma receptor interacting protein kinase-3 levels are associated with acute respiratory distress syndrome in sepsis and trauma: a cohort study
Source: Crit Care. 2019 Jun 28;23:235. doi: 10.1186/s13054-019-2482-x (PMC6599265; doi:10.1186/s13054-019-2482-x)

**Supplemental Figure 1**

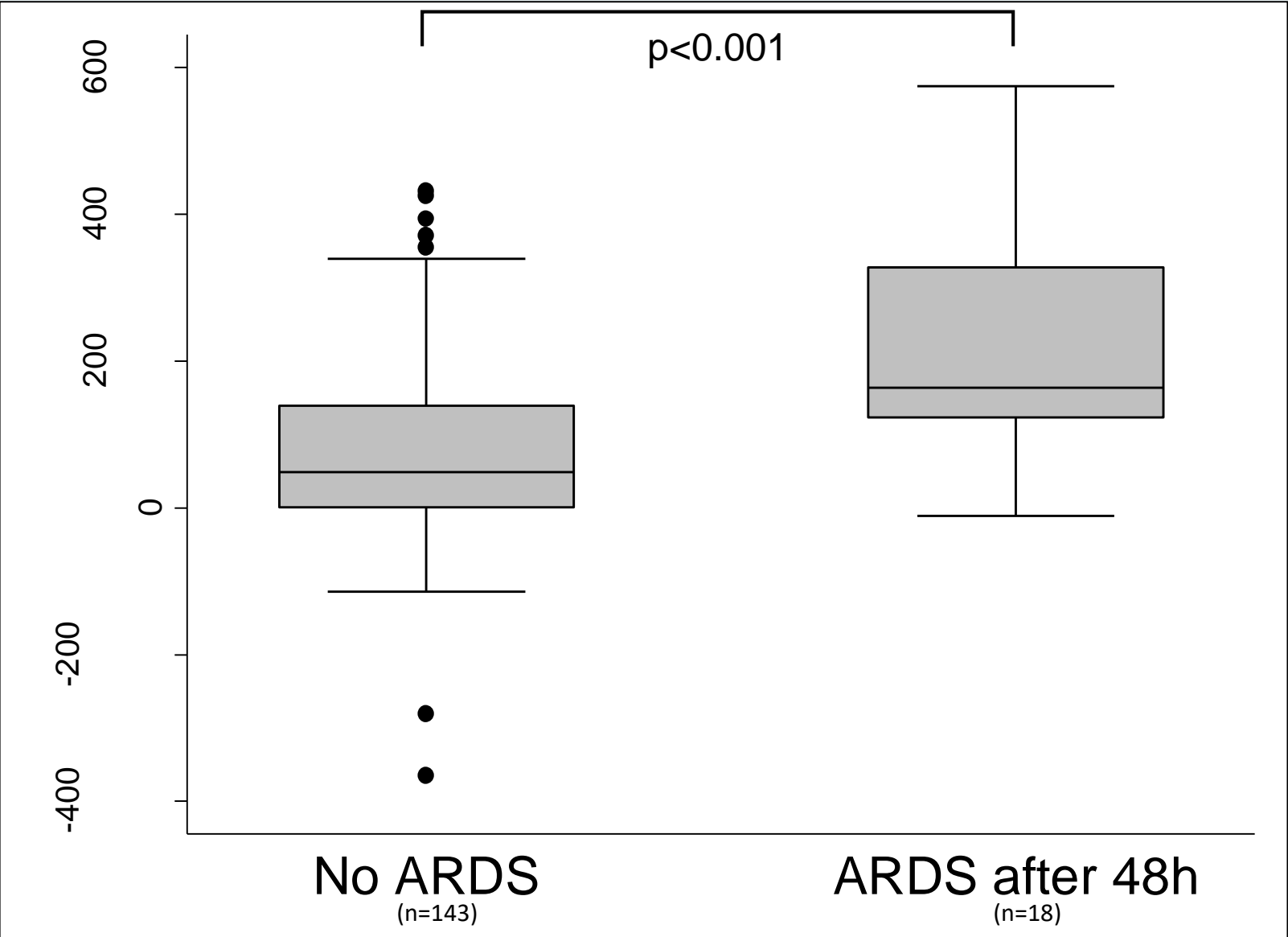

Supplement: Supplementary file 2 — Figure S1. Change in plasma RIPK3 concentration from presentation to 48 h by Acute Respiratory Distress Syndrome (ARDS) status excluding patients who met ARDS criteria ≤ 48 h after presentation among patients in the PETROS (trauma) cohort (No ARDS n = 143, ARDS n = 18 meeting criteria 55–140 h after presentation). Gray boxes represent interquartile range, with median designated by central line. Figure S2. Levels of mixed lineage kinase domain-like protein (MLKL) and phosphorylated MLKL (pMLKL) in whole lung homogenate are not significantly different between mice treated with ZVAD, LPS, or LPS-ZVAD. A. Immunoblot of whole lung homogenate showing similar MLKL and pMLKL regardless of treatment. B. Densitometry of whole lung homogenate. All comparisons between treatment groups are non-significant. Figure S3. Adjusted probability of acute kidney injury (AKI) across range of Δ receptor interacting protein kinase-3 (RIPK3) levels (RIPK3 change from presentation to 48 h) in each cohort. Estimated probabilities (line) with 95% confidence intervals (gray shading) determined using post-estimation marginal analysis after multivariable logistic regression modeling. A. MESSI cohort. Probabilities adjusted for age, red blood cell transfusions on the day of presentation, chronic kidney disease, diabetes mellitus, and shock at presentation. B. PETROS cohort. Probabilities adjusted for red blood cell transfusions in the first 6 h after presentation, trauma mechanism, abdominal injury severity, and shock prior to ICU admission. Figure S4. Plasma levels of Δ receptor interacting protein kinase-3 (ΔRIPK3, change from presentation to 48 h) by acute kidney injury (AKI) and acute respiratory distress syndrome (ARDS) category in human cohorts. Shaded portions of box plots show median concentrations (central line) and 25th and 75th percentiles (bottom and top lines of box). Brackets above the p values denote the two organ dysfunction categories being compared (Wilcoxon rank-sum test). A. MES [file 13054_2019_2482_MOESM2_ESM.zip › Supplemental Figure 1 ARDS after 48h.pdf]

# Supplemental Figure 2

A.

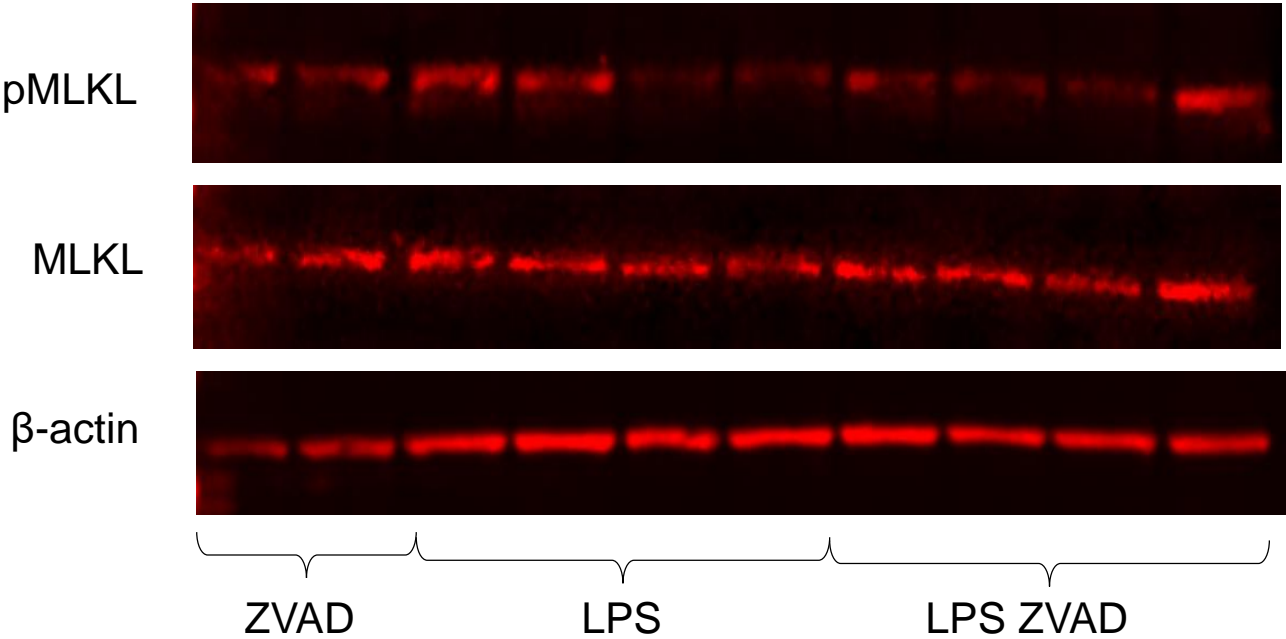

B.

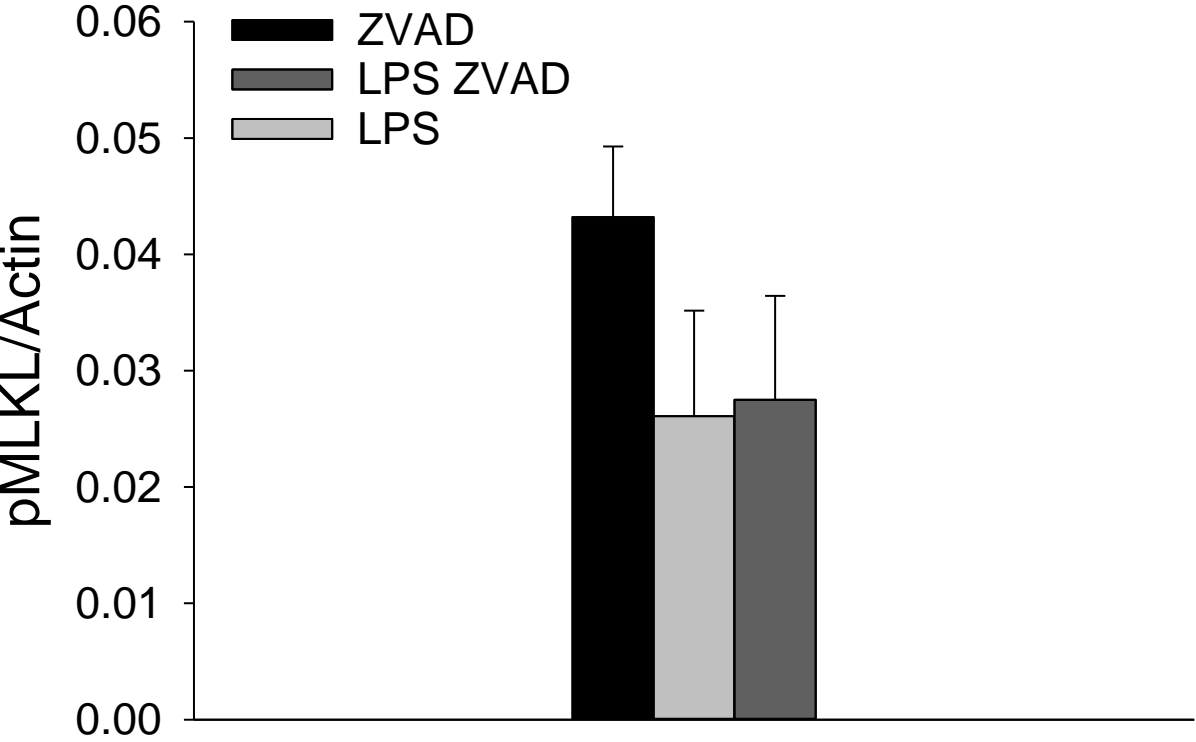

Supplement: Supplementary file 2 — Figure S1. Change in plasma RIPK3 concentration from presentation to 48 h by Acute Respiratory Distress Syndrome (ARDS) status excluding patients who met ARDS criteria ≤ 48 h after presentation among patients in the PETROS (trauma) cohort (No ARDS n = 143, ARDS n = 18 meeting criteria 55–140 h after presentation). Gray boxes represent interquartile range, with median designated by central line. Figure S2. Levels of mixed lineage kinase domain-like protein (MLKL) and phosphorylated MLKL (pMLKL) in whole lung homogenate are not significantly different between mice treated with ZVAD, LPS, or LPS-ZVAD. A. Immunoblot of whole lung homogenate showing similar MLKL and pMLKL regardless of treatment. B. Densitometry of whole lung homogenate. All comparisons between treatment groups are non-significant. Figure S3. Adjusted probability of acute kidney injury (AKI) across range of Δ receptor interacting protein kinase-3 (RIPK3) levels (RIPK3 change from presentation to 48 h) in each cohort. Estimated probabilities (line) with 95% confidence intervals (gray shading) determined using post-estimation marginal analysis after multivariable logistic regression modeling. A. MESSI cohort. Probabilities adjusted for age, red blood cell transfusions on the day of presentation, chronic kidney disease, diabetes mellitus, and shock at presentation. B. PETROS cohort. Probabilities adjusted for red blood cell transfusions in the first 6 h after presentation, trauma mechanism, abdominal injury severity, and shock prior to ICU admission. Figure S4. Plasma levels of Δ receptor interacting protein kinase-3 (ΔRIPK3, change from presentation to 48 h) by acute kidney injury (AKI) and acute respiratory distress syndrome (ARDS) category in human cohorts. Shaded portions of box plots show median concentrations (central line) and 25th and 75th percentiles (bottom and top lines of box). Brackets above the p values denote the two organ dysfunction categories being compared (Wilcoxon rank-sum test). A. MES [file 13054_2019_2482_MOESM2_ESM.zip › Supplemental Figure 2 lung MLKL LPS mouse.pdf]

# Supplemental Figure 3

## MESSI Cohort

A.

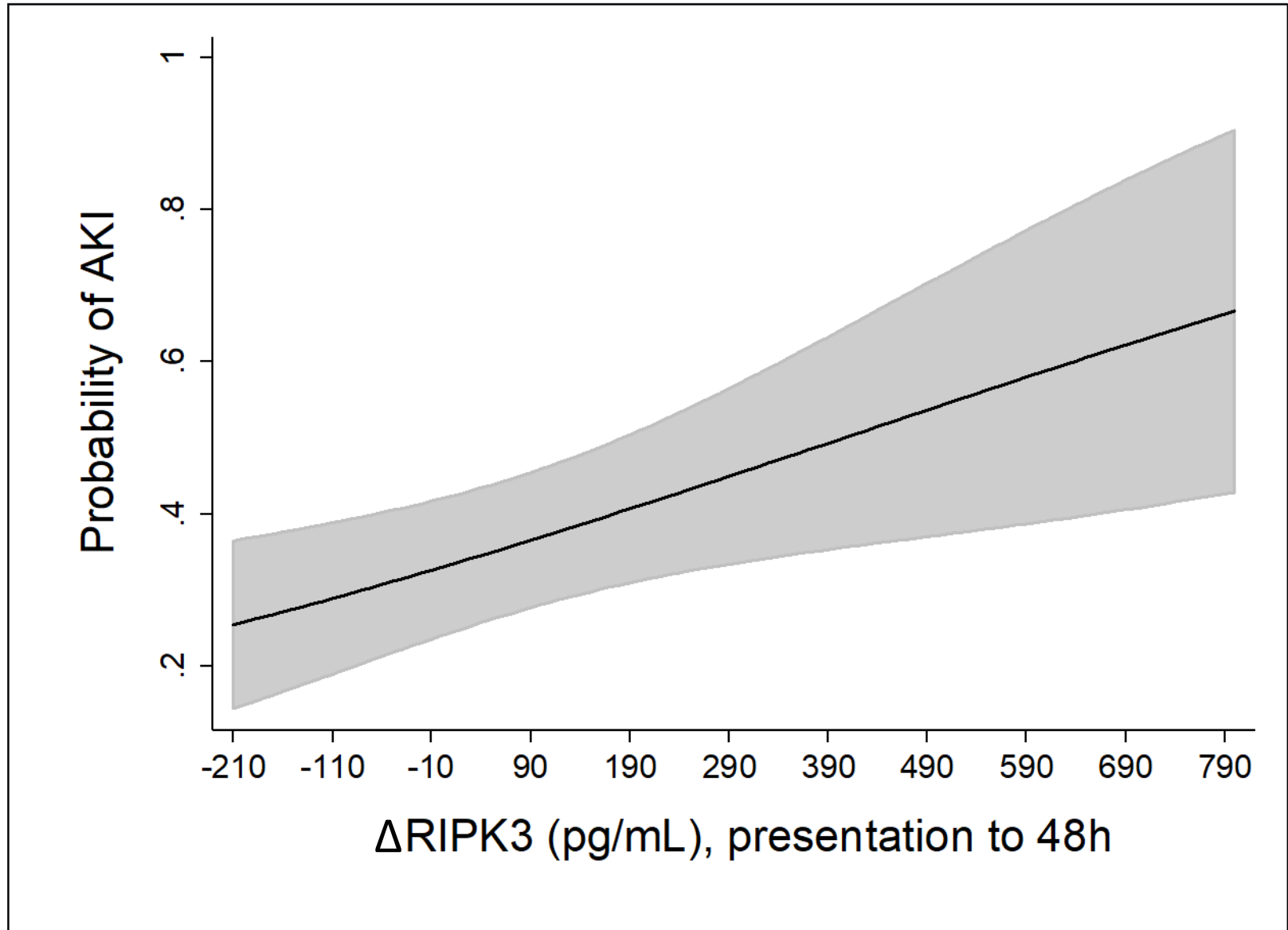

## PETROS Cohort

B.

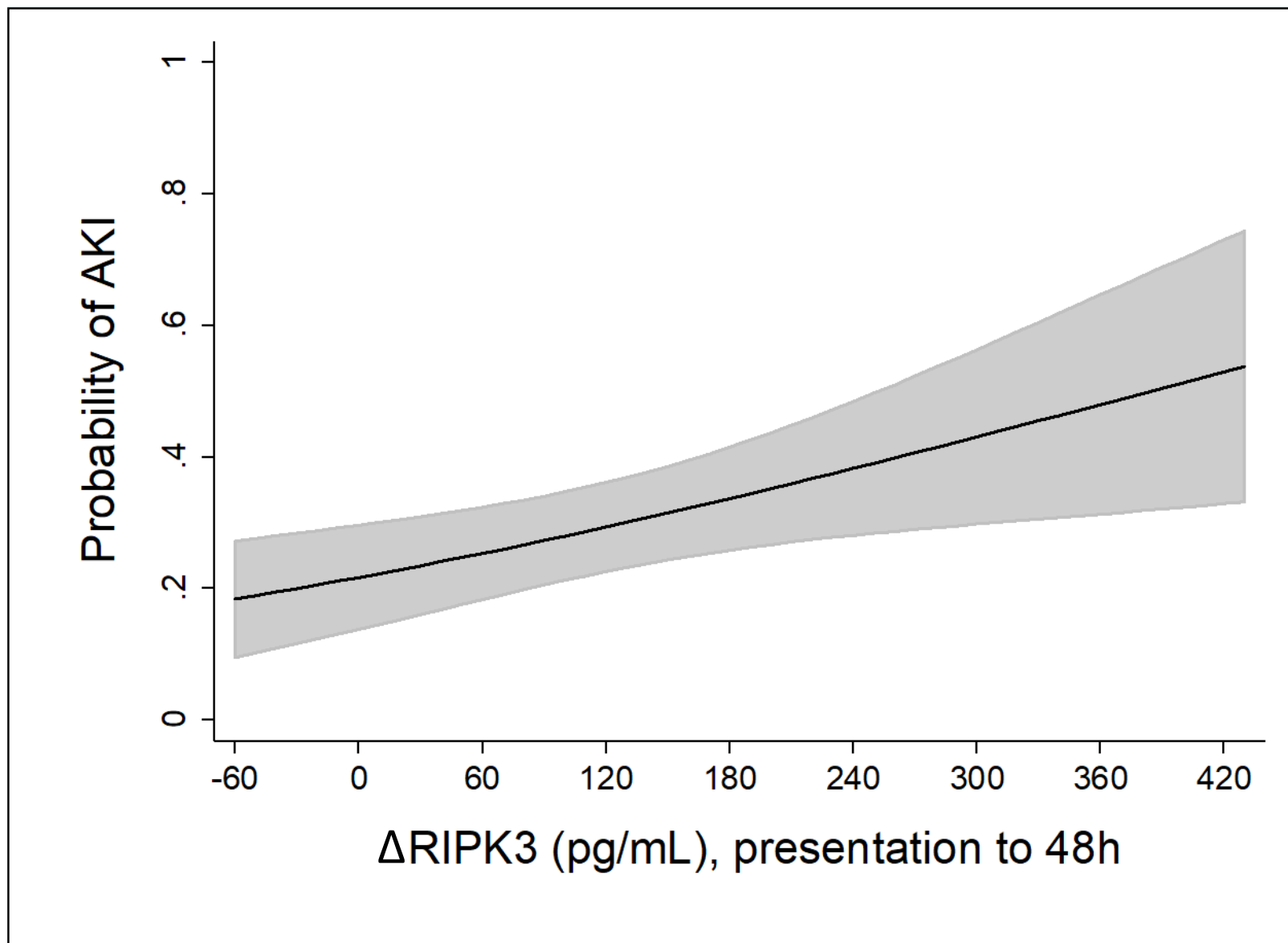

Supplement: Supplementary file 2 — Figure S1. Change in plasma RIPK3 concentration from presentation to 48 h by Acute Respiratory Distress Syndrome (ARDS) status excluding patients who met ARDS criteria ≤ 48 h after presentation among patients in the PETROS (trauma) cohort (No ARDS n = 143, ARDS n = 18 meeting criteria 55–140 h after presentation). Gray boxes represent interquartile range, with median designated by central line. Figure S2. Levels of mixed lineage kinase domain-like protein (MLKL) and phosphorylated MLKL (pMLKL) in whole lung homogenate are not significantly different between mice treated with ZVAD, LPS, or LPS-ZVAD. A. Immunoblot of whole lung homogenate showing similar MLKL and pMLKL regardless of treatment. B. Densitometry of whole lung homogenate. All comparisons between treatment groups are non-significant. Figure S3. Adjusted probability of acute kidney injury (AKI) across range of Δ receptor interacting protein kinase-3 (RIPK3) levels (RIPK3 change from presentation to 48 h) in each cohort. Estimated probabilities (line) with 95% confidence intervals (gray shading) determined using post-estimation marginal analysis after multivariable logistic regression modeling. A. MESSI cohort. Probabilities adjusted for age, red blood cell transfusions on the day of presentation, chronic kidney disease, diabetes mellitus, and shock at presentation. B. PETROS cohort. Probabilities adjusted for red blood cell transfusions in the first 6 h after presentation, trauma mechanism, abdominal injury severity, and shock prior to ICU admission. Figure S4. Plasma levels of Δ receptor interacting protein kinase-3 (ΔRIPK3, change from presentation to 48 h) by acute kidney injury (AKI) and acute respiratory distress syndrome (ARDS) category in human cohorts. Shaded portions of box plots show median concentrations (central line) and 25th and 75th percentiles (bottom and top lines of box). Brackets above the p values denote the two organ dysfunction categories being compared (Wilcoxon rank-sum test). A. MES [file 13054_2019_2482_MOESM2_ESM.zip › Supplemental Figure 3 RIP3-AKI margins plot.pdf]

## Supplemental Figure 4

### A. MESSI Cohort

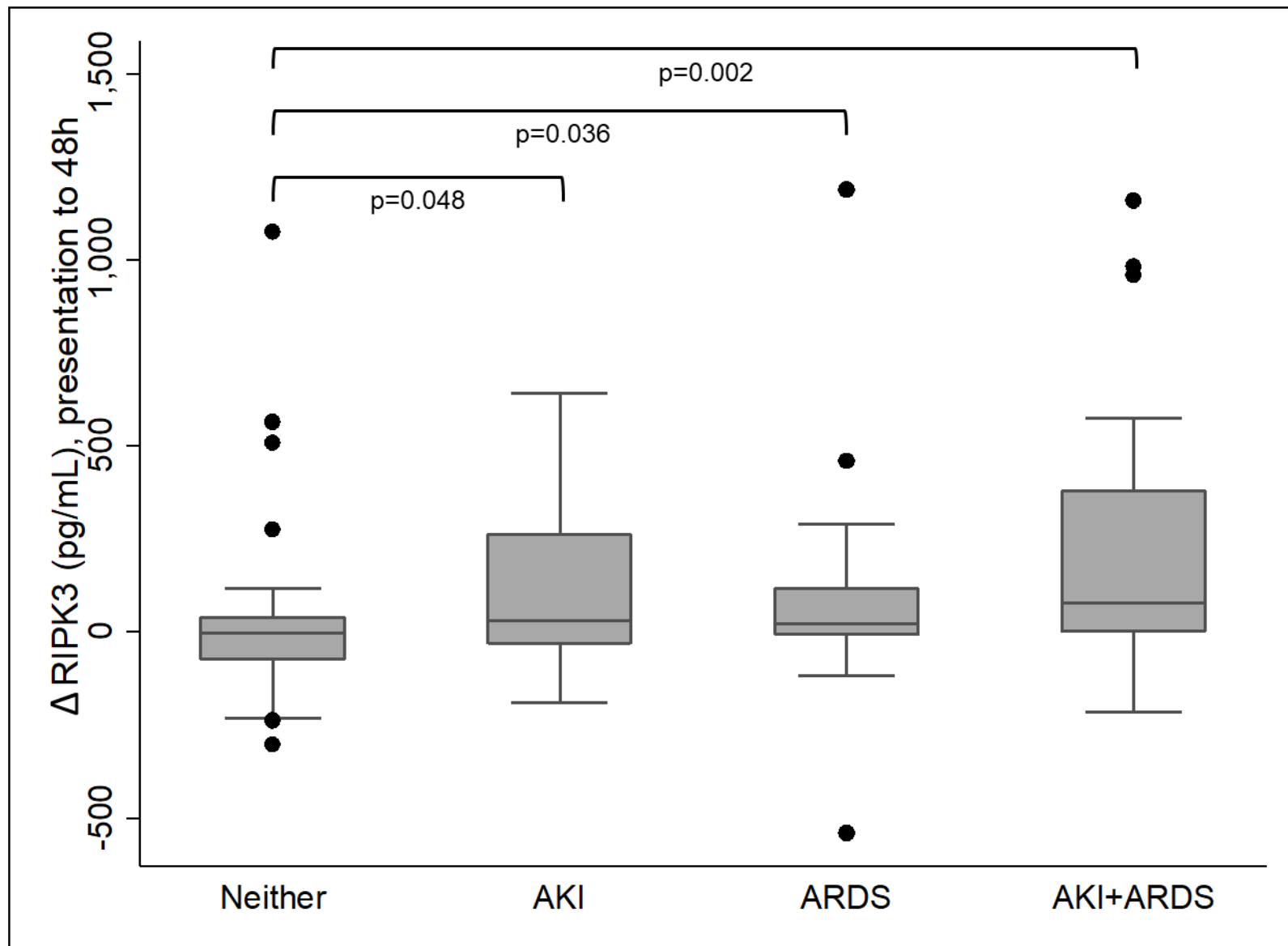

**B.**

## PETROS Cohort

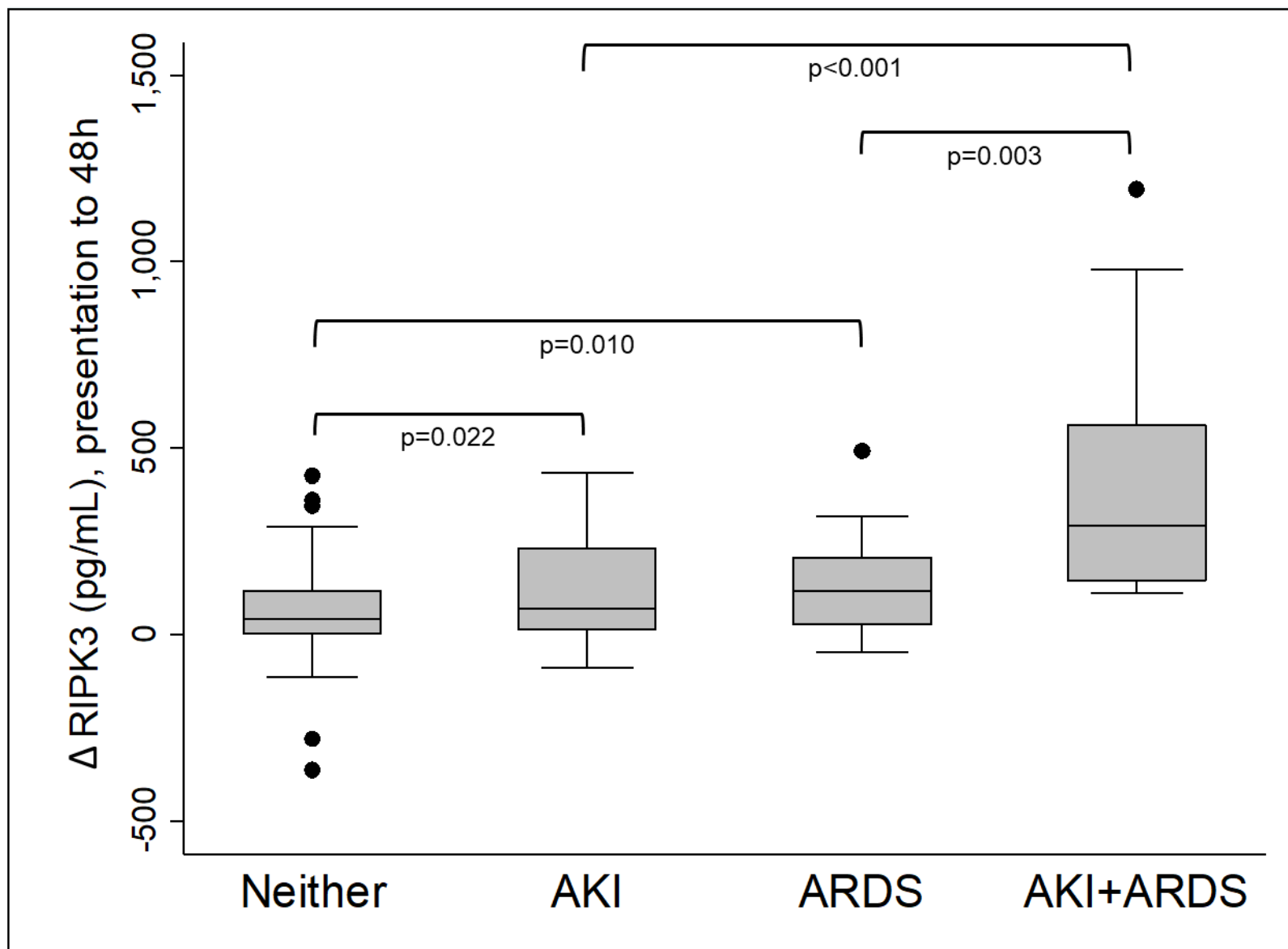

Supplement: Supplementary file 2 — Figure S1. Change in plasma RIPK3 concentration from presentation to 48 h by Acute Respiratory Distress Syndrome (ARDS) status excluding patients who met ARDS criteria ≤ 48 h after presentation among patients in the PETROS (trauma) cohort (No ARDS n = 143, ARDS n = 18 meeting criteria 55–140 h after presentation). Gray boxes represent interquartile range, with median designated by central line. Figure S2. Levels of mixed lineage kinase domain-like protein (MLKL) and phosphorylated MLKL (pMLKL) in whole lung homogenate are not significantly different between mice treated with ZVAD, LPS, or LPS-ZVAD. A. Immunoblot of whole lung homogenate showing similar MLKL and pMLKL regardless of treatment. B. Densitometry of whole lung homogenate. All comparisons between treatment groups are non-significant. Figure S3. Adjusted probability of acute kidney injury (AKI) across range of Δ receptor interacting protein kinase-3 (RIPK3) levels (RIPK3 change from presentation to 48 h) in each cohort. Estimated probabilities (line) with 95% confidence intervals (gray shading) determined using post-estimation marginal analysis after multivariable logistic regression modeling. A. MESSI cohort. Probabilities adjusted for age, red blood cell transfusions on the day of presentation, chronic kidney disease, diabetes mellitus, and shock at presentation. B. PETROS cohort. Probabilities adjusted for red blood cell transfusions in the first 6 h after presentation, trauma mechanism, abdominal injury severity, and shock prior to ICU admission. Figure S4. Plasma levels of Δ receptor interacting protein kinase-3 (ΔRIPK3, change from presentation to 48 h) by acute kidney injury (AKI) and acute respiratory distress syndrome (ARDS) category in human cohorts. Shaded portions of box plots show median concentrations (central line) and 25th and 75th percentiles (bottom and top lines of box). Brackets above the p values denote the two organ dysfunction categories being compared (Wilcoxon rank-sum test). A. MES [file 13054_2019_2482_MOESM2_ESM.zip › Supplemental Figure 4 RIP3-AKI-ARDS unadjusted.pdf]
